# Genome-Wide Association Studies Identify an Association of Transferrin Binding Protein B Variation and Invasive Serogroup Y Meningococcal Disease in Older Adults

**DOI:** 10.1093/infdis/jiac430

**Published:** 2022-11-02

**Authors:** Laura Maynard-Smith, Jeremy P Derrick, Ray Borrow, Jay Lucidarme, Martin C J Maiden, Robert S Heyderman, Odile B Harrison

**Affiliations:** Division of Infection and Immunity, University College London, London, United Kingdom; Faculty of Biology, Medicine, and Health, University of Manchester, Manchester, United Kingdom; Meningococcal Reference Unit, UK Health Security Agency, Manchester, United Kingdom; Meningococcal Reference Unit, UK Health Security Agency, Manchester, United Kingdom; Department of Biology, University of Oxford, Oxford, United Kingdom; Division of Infection and Immunity, University College London, London, United Kingdom; Department of Biology, University of Oxford, Oxford, United Kingdom

**Keywords:** invasive meningococcal disease, meningococcal serogroup Y, *Neisseria meningitidis*, transferrin binding protein B

## Abstract

**Background:**

*Neisseria meningitidis* serogroup Y, especially ST-23 clonal complex (Y:cc23), represents a larger proportion of invasive meningococcal disease (IMD) in older adults compared to younger individuals. This study explored the meningococcal genetic variation underlying this association.

**Methods:**

Maximum-likelihood phylogenies and the pangenome were analyzed using whole-genome sequence (WGS) data from 200 Y:cc23 isolates in the *Neisseria* PubMLST database. Genome-wide association studies (GWAS) were performed on WGS data from 250 Y:cc23 isolates from individuals with IMD aged ≥65 years versus < 65 years.

**Results:**

Y:cc23 meningococcal variants did not cluster by age group or disease phenotype in phylogenetic analyses. Pangenome comparisons found no differences in presence or absence of genes in IMD isolates from the different age groups. GWAS identified differences in nucleotide polymorphisms within the transferrin-binding protein B (*tbpB*) gene in isolates from individuals ≥65 years of age. TbpB structure modelling suggests these may impact binding of human transferrin.

**Conclusions:**

These data suggest differential iron scavenging capacity amongst Y:cc23 meningococci isolated from older compared to younger patients. Iron acquisition is essential for many bacterial pathogens including the meningococcus. These polymorphisms may facilitate colonization, thereby increasing the risk of disease in vulnerable older people with altered nasopharyngeal microbiomes and nutritional status.

## Teaser

Nucleotide polymorphisms in *tbpB* may differentially affect iron scavenging capacity among Y:cc23 meningococci isolated from older and younger patients. Y:cc23 represents a larger proportion of disease in older people, and these polymorphisms may facilitate colonization and disease in this population.

There are 12 different capsular polysaccharides of *Neisseria meningitidis*, of which serogroups A, B, C, W, X, and Y are associated with invasive meningococcal disease (IMD). In the United Kingdom, the highest incidence of IMD is in children and adolescents, with the majority caused by serogroup B (66% of all cases in England 2019–2020) [1]. In older adults, however, serogroups W and Y account for a much larger proportion of IMD with, for example, 22% (28/126) of cases in ≥65 year olds caused by serogroup Y meningococci in 2018–2019 in contrast to 3% (6/185) in under 15 year olds [1]. Such disparities in the age distribution of IMD due to serogroup Y have also been observed in Europe with 50% of cases occurring in 45–88 year olds [2]. Data from Sweden demonstrated that between 1995 and 2012, the median age of presentation with serogroup Y IMD was 62 years old. It is noteworthy that the age of presentation differed by disease etiology, with the median age of those presenting with meningitis, arthritis, or pneumonia being 22.5, 72, and 77.5 years, respectively [3].

In the United States during the 1990s, an increase in the incidence of serogroup Y IMD was mostly attributed to meningococci belonging to ST-23 clonal complex (cc23, Y:cc23), with the proportion of IMD overall caused by serogroup Y meningococci rising from 2% in 1989–1991 to 32.6% in 1996 [4]. A multivariate model of those with known serogroups from a US laboratory surveillance study showed that patients with serogroup Y IMD were more likely to be older (age >17 years; relative risk 1.4, *P* = .0002) [4]. This increase corresponded with an antigenic shift in the outer membrane proteins PorA, PorB, and FetA, consistent with immune selective pressure and immune escape [5]. Whole-genome sequence (WGS) analyses of the existing early and emerging late Y:cc23 variants also revealed differences in genes associated with pilin structure and iron acquisition [6]. This increase in Y:cc23 IMD in the United States was mirrored in Europe in the 2000s, with over 50% of IMD cases in Sweden in 2013 caused by serogroup Y [7]. In Sweden, WGS demonstrated that this increase was associated with expansion of Y:cc23 meningococci referred to as Y1 subtype 1, which were closely related to, but distinct from, theY:cc23 meningococci that caused IMD in the United States. The expansion of the Swedish strain was not, however, associated with major antigenic changes, suggesting that other unknown factors may have led to the increased prevalence of this meningococcal variant [8].

It is unclear why serogroup Y accounts for a greater proportion of IMD in older people. IMD is a complex phenotype that is a multifactorial combination of host and meningococcal genetic determinants, which may influence nasopharyngeal colonization and IMD progression in certain meningococci and age groups. For example, heterogeneity in the ability of different meningococcal serogroups to activate the alternative complement pathway through C3 activation has been documented [9]. In addition, such genetic determinants may not solely be due to presence and/or absence, given that both carriage and commensal *Neisseria* species possess many genes implicated in virulence [10, 11]. Host factors such as waning mucosal and systemic immunity, chronic inflammation, and dysregulated metabolism are associated with aging [12, 13]. Complement protein levels and the associated regulatory proteins, such as factor H and factor I, are also known to increase with age from neonates to adulthood, which may additionally contribute to age-related differences in IMD [9, 14].

Here, we have examined WGS data belonging to Y:cc23 isolates to identify the bacterial factors associated with IMD in older people. Hierarchical analyses were performed including genome comparisons through a gene-by-gene approach and genome-wide association studies (GWAS). Results from this study suggest that polymorphisms within genes affecting iron metabolism are implicated in the complex age-dependent meningococci-host interactions and could result in different Y:cc23 IMD disease rates in different age groups.

## METHODS

### Genomes

WGS data for serogroup Y meningococci were chosen from the publicly available *Neisseria* PubMLST web-accessible database (http://pubmlst.org/neisseria). Capsule group Y was defined genotypically, or phenotypically if the *csy* gene was incomplete. Isolates have been deposited in PubMLST from all over the world, and no restrictions are placed on where or when isolates are collected. To assess the impact of age on disease incidence, patient age data were obtained for UK invasive isolates from the UK Health Security Agency (UKHSA) Meningococcal Reference Unit, Manchester, which deposits the UK invasive genomes within PubMLST. These data were not available for the other isolates within PubMLST. Genomes deposited in the GenBank and European Nucleotide Archive databases did not have the necessary accompanying metadata, such as date or location of collection, or whether isolates were invasive or carried.

### Genome Comparison

PubMLST.org is implemented with the Bacterial Isolate Genome Sequence Database (BIGSdb) platform and uses a gene-by-gene approach to compare allele sequences stored at each locus to generate an allele identification number and subsequent genome-wide multilocus profile for isolates with whole-genome data [15]. One isolate representing each of the 206 different sequence types found within all the Y genomes in PubMLST were compared (total n = 206; Supplementary Table 1). The genome comparator tool was used to create a distance matrix based on the number of allelic differences across the 1605 genes within the *N. meningitidis* core genome (*N. meningitidis* cgMLST version 1.0) [16]. This was resolved into phylogenetic networks using the Neighbor-Net algorithm within SplitsTree4 (version 4.14.8).

A representative dataset of 250 genomes from all the available cc23 invasive and carried isolates were processed by the genome comparator tool to produce a concatenated whole-genome coding sequence alignment. Isolates were chosen on the basis of their being identified in the database as originating from carriage denoted with the “carrier” label, or invasive denoted by the labels “invasive (unspecified/other),” “meningitis,” “septicemia,” or “meningitis and septicemia.” An unrooted maximum likelihood phylogeny was reconstructed using PhyML with the HKY85 nucleotide substitution model [17], and then modified with ClonalFrameML, under the standard model with 100 simulations, -emsim 100 [18], to account for recombination. The tree was then annotated using iTOL (version 5) [19]. Testing with ClonalFrameML using increasing dataset sizes indicated that the analysis of a maximum of 250 genomes was achievable within the computational limits of this tool (Supplementary Table 2).

### Genome-Wide Association Studies

WGS data were downloaded in fasta format from 250 Y:cc23 IMD isolates from England dating from 2010 to 2017, 125 each from people aged <65 or ≥65 years (Supplementary Table 2). The patient age data were obtained from the UKHSA Meningococcal Reference Unit, which also made the genomes publicly available in the PubMLST.org/neisseria database for UK invasive isolates. Annotated assemblies of these genomes were produced by Prokka in GFF3 format and used to calculate the pangenome using Roary [20, 21]. A phylogenetic tree was constructed using fasttree [22]. Roary plots was used to visualize the tree compared to a matrix with the presence and absence of core and accessory genes. A pangenome-wide association analysis was performed by Scoary using the gene presence and absence output from Roary and age as the phenotypic trait of interest to score components of the pangenome as having an association [23].

Meningococcal nucleotide polymorphisms associated with disease in either <65 or ≥65 year olds were identified using TreeWas [24]. This employs a phylogenetic approach that avoids the confounding effects of population structure and recombination. It creates a simulated dataset, which resembles the empirical dataset in population structure and composition but comprises only potential confounding factors. The association between the simulated dataset and phenotype were then compared to the association between the empirical dataset and phenotype to test for true associations. Three separate tests were used to produce 3 scores: (1) the terminal score measured sample-wide association across the leaves of the phylogenetic tree; (2) the simultaneous score measured the degree of parallel change in the phenotype and genotype across branches of the tree; and (3) the subsequent score, which measured the proportion of the tree in which the genotype and phenotype coexist. A single-nucleotide polymorphism only need be significant in 1 of the 3 scores to be assumed to be significant overall. The TreeWas R package has been designed to work with the outputs of ClonalFrameML. A subset of 125 isolates from <65 and ≥65 year olds (250 per analysis) were analyzed using ClonalFrameML, with the outputs combined with the age-associated data to calculate the 3 scores and attached *P* values within TreeWas. The TreeWas output provided the location on the genome of the polymorphisms with the lowest probability, which were then mapped onto the alignment to identify the gene involved. A cutoff of <1 × 10^−4^ was considered to be significant. TreeWas analyses were carried out in triplicate using different subsets of 250 isolates each for reproducibility of results (Supplementary Table 2).

### Transferrin Binding Protein B Structural Analysis

Multiple sequence alignments of TbpB peptide isotype II variants were generated using the Multiple Alignment Using Fast Fourier Transform (MAFFT) program available on the European Bioinformatics Institute-European Molecular Biology Laboratory (EBI-EMBL) open access website using the translated gene sequence from meningococcal isolate M982 as a reference sequence (https://www.ebi.ac.uk/Tools/msa/mafft/). Residues were highlighted that corresponded to the polymorphisms identified by the TreeWas analysis within TbpB. A space-filling model and ribbon plot of TbpB were generated using CCP4MG [25] and used Protein Data Bank (PDB) code 3VE1 [26]. The residues identified by TreeWas analysis were highlighted.

## RESULTS

Within the PubMLST.org/neisseria database (accessed 4 February 2019), there were 2540 serogroup Y WGSs obtained from isolates dating from 1986 to 2019. Of these, 971 were carriage isolates (38%), 997 invasive isolates (39%), with the remainder “unspecified,” that is of unknown disease provenance (23%). Most isolates originated from the United Kingdom (1630/2540, 64%), with the next most common countries being Sweden (312/2540, 12%), France (136/2540, 5%), United States (98/2540, 4%), and South Africa (60/2540, 2%). Of the 2399 isolates with a clonal complex assigned, 1876 belonged to cc23 (78%). There were 206 different sequence types, with the most common ccs being cc23 (83/206, 40%); cc167 (28/206, 13%); cc22 (9/206; 4%); cc92 (7/206, 33%); cc103 (6/206, 3%); and cc174 (6/206, 3%).

### Age and Invasive Meningococcal Disease

**Figure 1.**
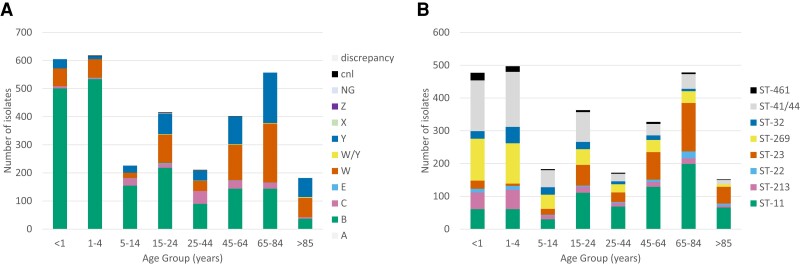
Invasive *Neisseria meningitidis* isolates by age group. *A,* Number of invasive isolates by serogroup. *B,* Number of invasive isolates by clonal complex. Age group, serogroup, and clonal complex data provided by the UK Health Security Agency Meningococcal Reference Unit for invasive isolates identified in England, 2009–2019. Only those clonal complexes with n > 50 isolates were included.

Patient age data were available for 3223 isolates from the UKHSA Meningococcal Reference Unit for IMD isolates obtained in England between 2009 and 2019. In adults aged ≥65 years, serogroup W was the most common serogroup (278/741, 38%) followed by serogroup Y (247/741, 33%). In those where the clonal complex was known, cc23 accounted for 29% of all cases in ≥65 year olds (199/682) and in 87% of all serogroup Y cases (195/223), but only 7% of all cases in those aged 24 years and younger (113/1664) (Figure 1).

### Genome Comparison

**Figure 2.**
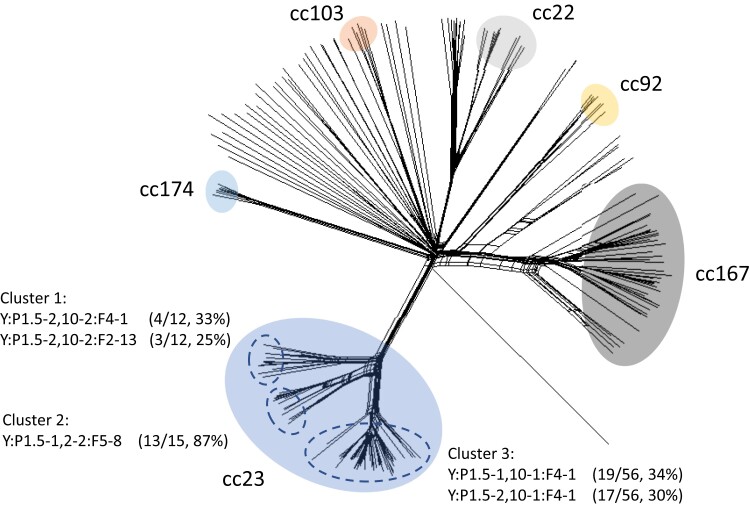
Neighbor-Net genome comparison of *Neisseria meningitidis* serogroup Y. Neighbor-Net Tree comparing allelic profiles of 1605 loci core to the meningococcal genome (*N. meningitidis* cgMLST version 1.0) belonging to 206 serogroup Y meningococci, representative of each sequence type within the global collection of serogroup Y isolates deposited in the PubMLST database. Clonal complexes that consist of more than 5 separate sequence types are labelled. The most common strain designations found in the 3 cc-23 subclusters are indicated.

A phylogenetic network reconstructed with the Neighbor-Net algorithm identified Y:cc23 isolates as being distinct from the remaining serogroup Y-associated clonal complexes, differentiating into 3 smaller clusters (Figure 2). Each smaller Y:cc23 cluster was characterized by distinct fine types, defined by serogroup, PorA, and Fet A variant and clonal complex, but these were not correlated with year of isolation or geographical area (Supplementary Table 1).

**Figure 3.**
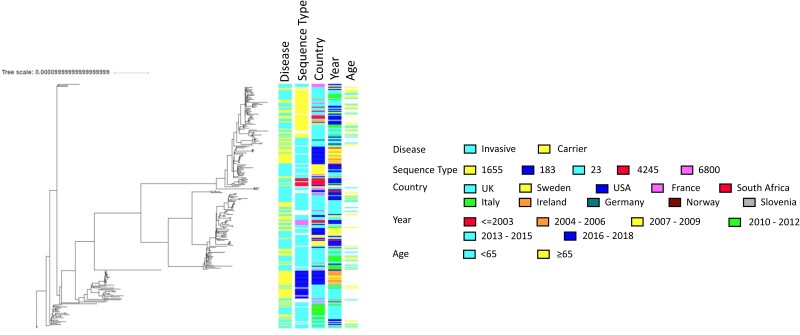
ClonalFrameML maximum likelihood tree of *Neisseria meningitidis* clonal complex cc-23. Analysis depicts 250 NmY genomes representative of cc23. Only those sequence types where n > 5 isolates were included.

A recombination-adjusted maximum likelihood tree of 250 representative Y:cc23 isolates was generated, which exhibited 5 monophyletic groups (Figure 3). There was little evidence for temporal or geographical clustering, nor was there any separation by whether they were invasive or carriage isolates. Age data were only available for UK invasive isolates, but isolates originating in people aged over or under 65 years were widely distributed in the phylogeny. Mapping sequence types onto the tree showed that ST-1655 and ST-183 were limited to monophyletic groups, in contrast to ST-23, which was widely distributed in the phylogeny.

### Genome-Wide Association Studies

**Figure 4.**
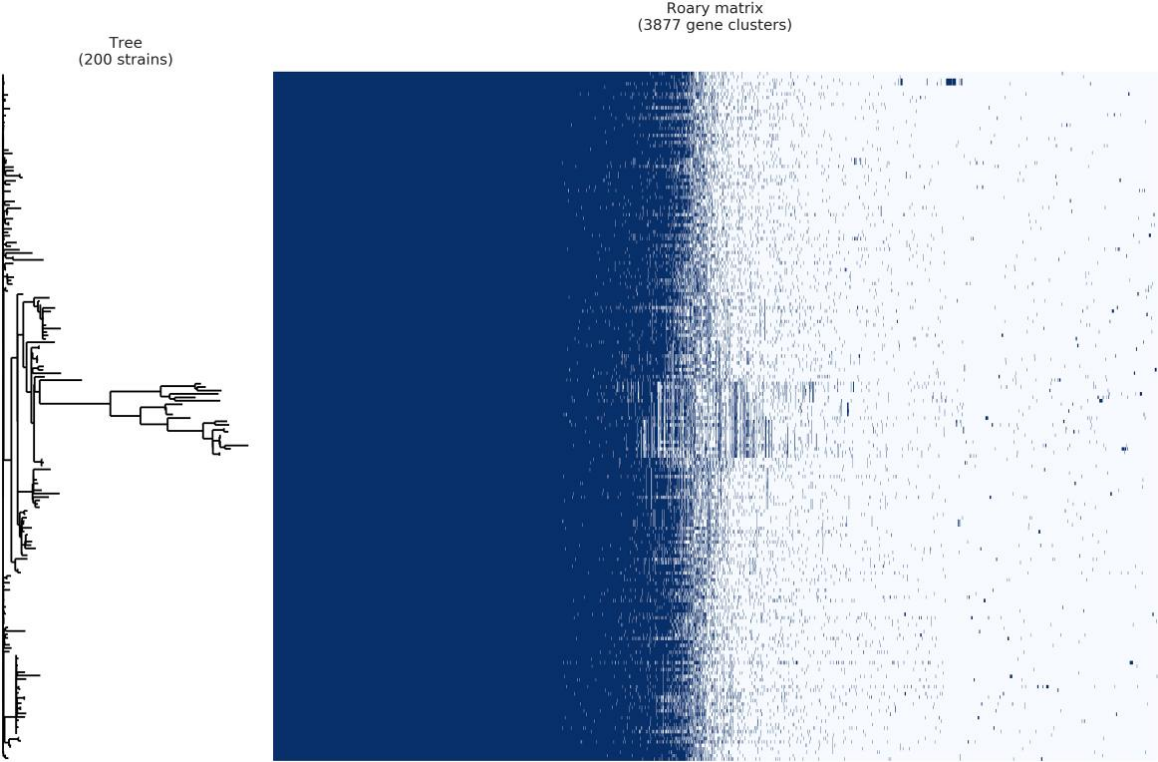
Roary plot of 200 *Neisseria meningitidis* cc23 isolates; 100 isolates originating from <65 year olds and 100 isolates from ≥65 year olds were chosen for phylogenetic analyses. Matrix depicts presence (dark) or absence (white) of genes across the pangenome in relation to the isolates within the phylogenetic tree. For invasive isolates identified in England, 2009–2019, the data provided by the UK Health Security Agency Meningococcal Reference Unit.

The pangenome for a subset of 200 Y:cc23 isolates (100 from people aged ≥65 years and 100 from <65 year olds) was calculated using Roary. This comprised 3877 genes in total, with the core genome comprising 1497 genes (39%). A matrix visualizing the presence and absence of genes in the accessory genome did not reveal associations within the phylogenetic tree (Figure 4). From the output of highest scoring genes, the majority were hypothetical proteins and none had both high sensitivity and specificity scores (Supplementary Table 3).

**Figure 5.**
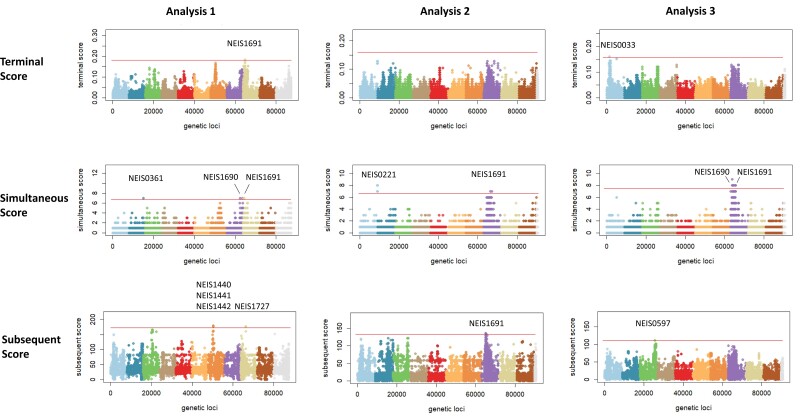
TreeWas plots of 250 *Neisseria meningitidis* cc23 isolates. Three analyses using a selection 250 cc23 NmY genomes each. TreeWas creates a simulated dataset that resembles the empirical dataset in population structure and composition comprising potential confounding factors and compares associations between both datasets and phenotype to test for true associations. Each TreeWas analysis provides 3 scores; significance level *P* < .0001 indicated by horizontal line. Terminal score measures sample-wide association across leaves of phylogenetic tree; simultaneous score measures degree of parallel change in phenotype and genotype across branches of tree; subsequent score measures proportion of tree in which genotype and phenotype coexist. Manhattan plot depicts individual nucleotide polymorphisms positioned along the x-axis according to chromosomal position (each such polymorphism is a separate dot; colors used to represent different regions of the chromosome) against the negative log of the single-nucleotide polymorphisms associated *P* value on the y-axis. Data provided by the UK Health Security Agency Meningococcal Reference Unit for invasive isolates identified in England, 2009–2019.

A TreeWas analysis was performed 3 times on a selection of 125 isolates from <65 year olds and 125 from ≥65 year olds to check reproducibility of results. Nucleotide polymorphisms within 10 genes were identified as significant (*P* < 1 × 10^−4^) (Supplementary Table 4). Twenty-four nonsynonymous polymorphisms in transferrin binding protein B (NEIS1691) were identified across the 3 analyses overall with a score that was significant (Figure 5). Transferrin binding protein A (NEIS1690) was also identified in 2 out of the 3 analyses, but these were both synonymous. The gene with the second most nonsynonymous mutations identified encodes MafI (NEIS0221), an immunity protein within the multiple adhesin family (7 identified).

### Location of *tbpb* Polymorphisms and Implications for Transferrin Binding

**Figure 6.**
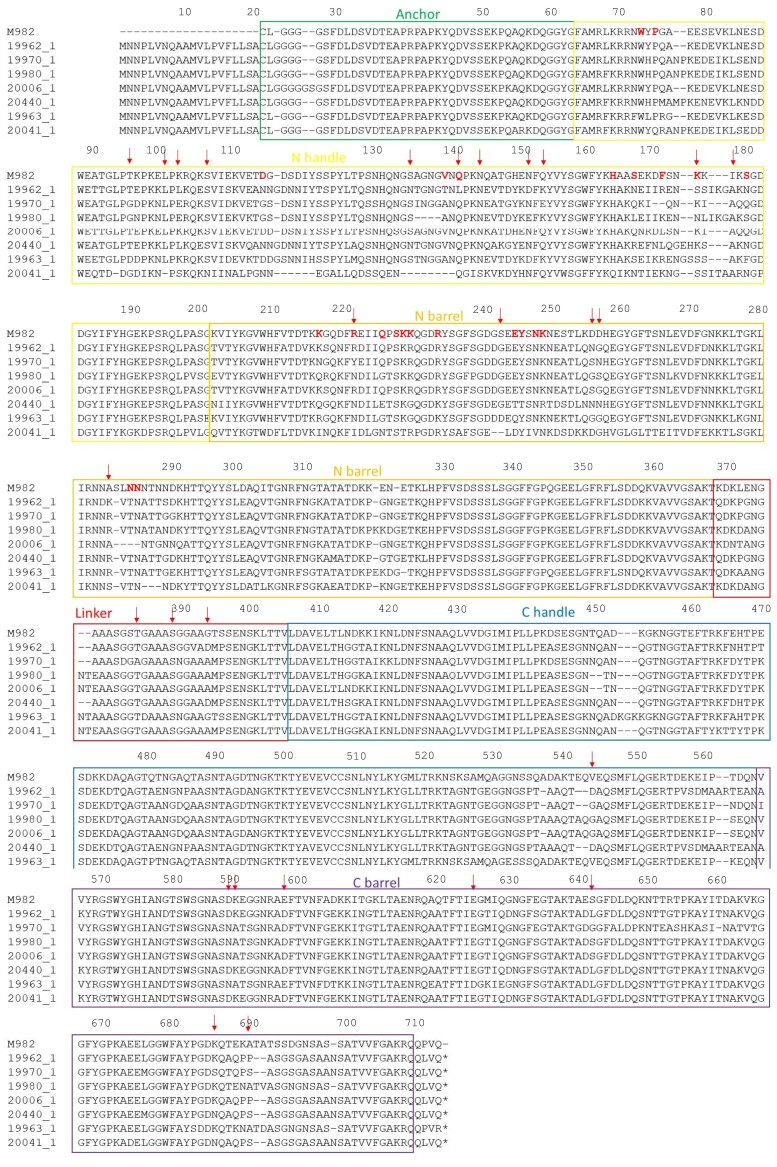
Transferrin binding protein B isotype II peptide sequence alignment. Reference sequence M982 and 7 other isolates with differing tbpB alleles. Red bold letters indicate previously published interactions between reference sequence and human transferrin. Arrows indicate polymorphisms found from the TreeWas analyses (24 in total).

**Figure 7.**
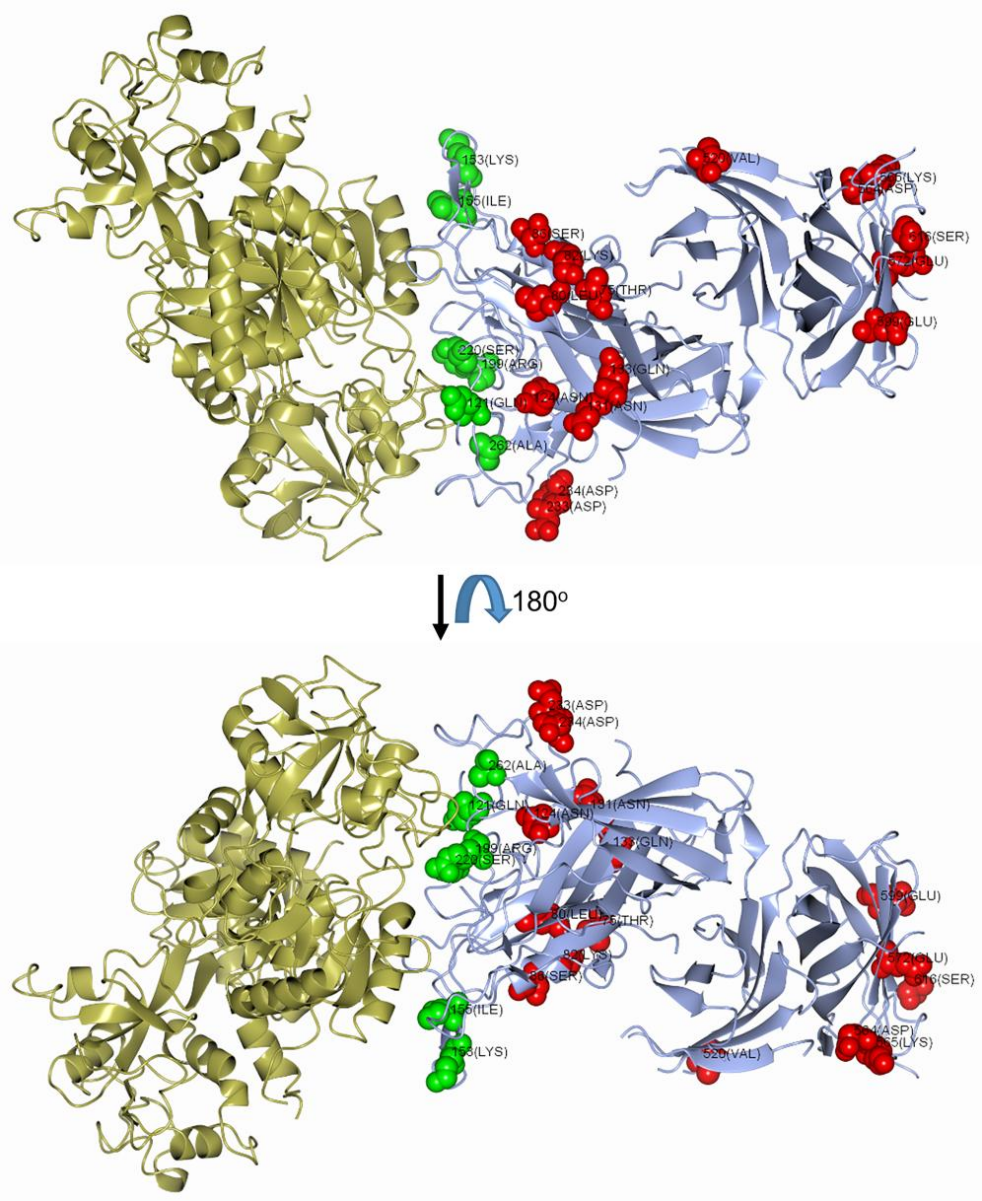
Transferrin binding protein B structure. Two views of the complex are shown, separated by a rotation of approximately 180^o^ around the horizontal axis. Human transferrin is shown in gold and TbpB in blue. Residues identified by TreeWas analysis as subject to polymorphism (Figure 6), and close to transferrin, are shown as spacefilling models in green. Other residues identified from the TreeWas analysis, but more remote from transferrin, are shown as spacefilling models in red. The figure was generated using CCP4MG [27] and used PDB code 3VE1 [28].

*N. meningitidis* isolates can be classified into 2 major *tbpB* families: isotype I (*tbpB* gene of 1.8 kb and TbpB protein of approximately 68 kDa) and isotype II (*tbpB* gene of 2.1 kb and TbpB protein with a mass of approximately 80 to 90 kDa) [27]. TbpB isotype II peptide sequences were aligned against a previously annotated reference sequence from meningococcal strain M982 obtained from the EBI-EMBL database. The majority of the polymorphisms (16/24, 67%) identified from the TreeWas analysis clustered in the part of *tbpB* encoding N-terminal region of TbpB, close to where interactions occur with human transferrin and TbpA. There were 8 further polymorphisms causing substitutions in the C-handle and barrel regions of the TbpB protein (Figure 6 and Figure 7). Those closest to the TbpB:transferrin binding interface were Q121, K153, I155, R199, S220, and A262 (using the sequence from the PDB deposition 3VE1) [26]. Of these, substitutions of R199 were the most likely to have an impact on transferrin recognition. R199 forms a salt bridge with E625 in transferrin, which would be removed with noncharged substitutions (Y/N/I) at this position. I155 forms part of a small hydrophobic pocket, which interacts with V360 on transferrin. Inspection of sequence alignments suggests that this is part of a relatively variable part of the TbpB sequence (Figure 6) and it is therefore difficult to predict possible effects of substitutions to K153 and I155 on transferrin affinity. A third point of contact with transferrin was Q121 to G502, which lies at the tip of a loop section on the transferrin surface. Q121 lies within a region of poor electron density within the crystal structure, suggesting that the TbpB chain is more flexible in this part of the TbpB structure and that mutations to Q121 are less likely to have an impact on transferrin binding affinity.

## DISCUSSION

Rates of IMD differ widely geographically and temporally, with certain meningococcal serogroup:clonal complex associations exhibiting differing rates of carriage and invasive disease. A number of meningococcal variants, the hyperinvasive lineages, are consistently more likely to cause IMD than others [28]. Serogroup Y meningococci, particularly Y:cc23 organisms, can be considered hyperinvasive and account for a higher proportion of IMD in adults aged ≥65 years in the United Kingdom than in younger cohorts [1]. The disease patterns are not uniform among this serogroup, for example, among serogroup Y IMD isolates obtained in England and Wales from 2007 to 2009, Y:cc23 were associated with meningitis in younger age groups (<25), whereas Y:cc174 meningococci were associated with nonmeningitis disease manifestations, particularly pneumonia, in older adults (≥65 years) [29]. Isolates included in the latter study [29] contained mutations in the *lpxL1* gene, which results in underacylated lipid A within the endotoxin lipooligosaccharide. In other studies of *lpxL1*, lipid A variants containing gene mutations that also reduced acylation of lipid A within serogroup B and C isolates were associated with disease in older age groups. IMD caused by such meningococci is more frequently associated with comorbidities, and with less severe infections, possibly due to reduced interaction with Toll-like receptor 4 (TLR4), when compared with the wild type [30].

The present study investigated whether genetic variation within Y:cc23 meningococci could account for differences in incidence among age groups. No differences were found in the presence or absence of genes within the pangenome of invasive isolates between the 2 age groups, nor did age cluster within phylogenetic trees. However, GWAS identified nonsynonymous polymorphisms predominantly within the gene encoding transferrin binding protein B (*tbpB*), and secondly within the gene encoding MafI, an immunity protein within the multiple adhesin family. The *maf* genes occur on genomic islands within pathogenic *Neisseria* species and encode a toxin-immunity system that is thought to confer an advantage in interbacterial competition among pathogenic *Neisseria* species [31].

The polymorphisms in TbpB were in regions that potentially impact transferrin uptake. In mammals, extracellular iron is tightly bound to iron-binding proteins, such as transferrin and lactoferrin, which act as a primary defense against invading pathogens by sequestering available iron. A wide range of evidence supports the idea that the acquisition of iron from host transferrin is essential for the survival and growth of *N. meningitidis* in the bloodstream and nasopharynx in both colonization and invasion. Many studies, dating from the 1970s, demonstrate the importance of iron acquisition in meningococcal disease [32], exemplified by the presence of multiple meningococcal ATP-dependent iron acquisition systems including transferrin binding proteins (TbpAB), lactoferrin binding proteins (LbpAB), and hemoglobin (HmbR) and hapto-hemoglobin receptors (HpuAB). One study using transgenic mice expressing human transferrin found amino acid substitutions were observed in several iron acquisition genes that were associated with differences in virulence capacity in mice between meningococci belonging to cc11 and cc23 [33]. In other studies of transgenic mice expressing human transferrin, *tbpB* mutants were cleared significantly more rapidly than wild-type meningococci [34], and in human experimental infection models, *tbpA/tbpB* deletion mutants of the closely related species *Neisseria gonorrhoeae* did not cause disease [35]. Furthermore, the growth of *tbpA*/*tbpB* mutant meningococci in the whole blood of healthy human donors is impaired compared to wild types [36].

TbpB is an anchored lipoprotein, which extends from the outer membrane surface and is involved in capturing the iron-loaded human transferrin. TbpA then acts to transport iron across the outer membrane [32]. The nonsynonymous polymorphisms identified using TreeWas clustered within regions of the TbpB protein that have been shown to interact with TbpA and human transferrin to enable iron scavenging [37]. In conclusion, it is plausible that certain amino acid substitutions may affect TbpB and its interactions with TbpA. Such mutations may in turn influence the ability of different meningococci to proliferate in individuals with differing iron availability.

Iron deficiency is the most common nutritional deficiency disorder in the world [38]. Specifically, in older people, insufficient dietary iron intake, malabsorption, and gastrointestinal blood losses all contribute to clinically relevant iron deficiencies [38, 39]. Iron status is often difficult to determine, but it has been estimated that around 10% of people aged ≥65 years are anemic, doubling in those ≥85 years old, and hemoglobin concentrations are known to decline with age, even in the absence of demonstrable disorders [40]. The differences in iron availability and microbiome in older age is consistent with the idea that meningococcal variants exhibiting different iron scavenging systems may differentially colonize hosts of different age. How changes in TbpB could enable adaptation to a changing microbiome and nutritional status, however, remain to be determined.

The upper respiratory tract microbiome changes with age and lifestyle factors such as smoking [41]. There is much less diversity in the microbiome of the anterior nares and oropharynx of the elderly compared to middle-aged adults and, although *Neisseria* species have been identified in the elderly [42], there is a paucity of large-scale meningococcal carriage studies in this age group. Nasopharyngeal carriage of meningococci is a prerequisite for disease, and it has been shown that the MenACWY vaccine has reduced the overall carriage of Y:cc23 among adolescents [43]. Although older adults do not have access to meningococcal vaccines as many of these form part of childhood immunization schedules, carriage of different clonal complexes could be indirectly affected in this population through herd immunity. The effect of this, exposure to different clonal complexes over time, and possible declining immunity with age could be addressed by larger-scale carriage studies in older people to establish why they are more at risk of Y:cc23 IMD than younger adults and children.

## CONCLUSION

In comparison with younger age groups, Y:cc23 meningococci cause a higher proportion of IMD in older adults. A GWAS analysis identified nonsynonymous nucleotide polymorphisms within the *tbpB* gene that differed between younger and older age groups. Iron acquisition is a key virulence mechanism and differences in iron scavenging between meningococcal variants may result in the differential colonization of individuals dependent on their age, nasopharyngeal microbiome, and nutritional status. This in turn could lead to age-related differences in the likelihood of colonized individuals developing IMD, providing a plausible mechanism for the well-established predilection of Y:cc23 meningococci to cause disease in older adults. By contrast, none of the other analyses conducted here identified systematic differences among meningococci that could explain this phenomenon.

## Supplementary Data

Supplementary materials are available at *The Journal of Infectious Diseases* online. Consisting of data provided by the authors to benefit the reader, the posted materials are not copyedited and are the sole responsibility of the authors, so questions or comments should be addressed to the corresponding author.

## Notes

***Acknowledgments.*** We thank the MRF Meningococcus Genome Library for providing genomes through the Neisseria MLST Database. This study made use of *Neisseria* genomic data deposited in the *Neisseria* PubMLST Database (https://pubmlst.org/neisseria/) sited at the University of Oxford [44]; the development of this database has been funded by the Wellcome Trust and European Union. We thank Dr Andrea Gori for his help in analyzing the sequence data.

***Disclaimer***. The findings and the views expressed are those of the authors and not necessarily those of the NIHR or any other affiliated institutions. The funders had no role in study design, data collection and analysis, decision to publish or preparation of the manuscript.

***Financial support.*** This work was supported by the National Institute for Health and Care Research (NIHR) (grant number ACF-2015-18-029 to L. M. S.); the Wellcome Trust (grant numbers 218205/Z/19/Z to M. C. J. M. and 214374/Z/18/Z to O. B. H.); and the Department of Health and Social Care using UK aid funding managed by the NIHR (grant number PR-OD-101720007 O. B. H.).

The NIHR Global Health Research Unit on Mucosal Pathogens was funded using UK aid from the UK Government (grant number 16/136/46 to R. S. H., a NIHR Senior Investigator).

## Supplementary Material

jiac430_Supplementary_Data
