# Supplementary material for: Genome-Wide Association Studies Identify an Association of Transferrin Binding Protein B Variation and Invasive Serogroup Y Meningococcal Disease in Older Adults
Source: J Infect Dis. 2022 Nov 2;226(12):2204–14. doi: 10.1093/infdis/jiac430 (PMC9748998; doi:10.1093/infdis/jiac430)
Supplement: jiac430_Supplementary_Data [file jiac430_supplementary_data.docx]

**Supplementary Table 1** List of serogroup Y isolates used in preliminary phylogenetic analyses (Figure 2). CC23:Y isolates are labelled by cc23 cluster.

| **PubMLST id** | **country** | **year** | **strain_designation** | **cc23 cluster** |
| --- | --- | --- | --- | --- |
| 661 | The Netherlands | 1986 | Y: P1.5-1,10-4: F4-1: ST-29 (cc167) |  |
| 930 | Czech Republic | 1993 | Y: P1.21,16: F4-5: ST-116 (cc116) |  |
| 934 | Czech Republic | 1993 | Y: P1.22,13-9: F1-5: ST-130 () |  |
| 966 | Czech Republic | 1993 | Y: P1.5-1,10-14: F5-14: ST-92 (cc92) |  |
| 971 | Czech Republic | 1993 | Y: P1.5-1,10-1: F1-5: ST-84 (cc92) |  |
| 1579 | Czech Republic | 1993 | Y: P1.5-1,10-1: F1-5: ST-734 (cc92) |  |
| 1581 | Czech Republic | 1993 | Y: P1.5-1,9: F1-5: ST-709 () |  |
| 1614 | Czech Republic | 1993 | Y: P1.5-1,10-4: F1-5: ST-94 (cc92) |  |
| 1648 | Czech Republic | 1993 | Y: P1.7-1,4-1: F1-5: ST-95 (cc92) |  |
| 8146 | Czech Republic | 1993 | Y: P1.12-3,4: F1-5: ST-133 (cc116) |  |
| 8148 | Czech Republic | 1993 | Y: P1.5-1,10-1: F1-5: ST-91 (cc92) |  |
| 15173 | Czech Republic | 1993 | Y: P1.18-1,3: F4-1: ST-36 () |  |
| 19976 | UK [England] | 2010 | Y: P1.5-2,10-2: F3-1: ST-9813 (cc23) | Cluster 1 |
| 19981 | UK [England] | 2010 | Y: P1.5-1,10-10: F4-1: ST-9814 (cc23) | Cluster 3 |
| 20135 | UK [England] | 2010 | Y: P1.5-1,10-1: F4-1: ST-9831 (cc23) | Cluster 3 |
| 20223 | UK [England] | 2011 | Y: P1.&Delta;,&Delta;: F3-4: ST-7786 () |  |
| 20227 | UK [England] | 2011 | Y: P1.21,16: F1-5: ST-9844 () |  |
| 20281 | UK [England] | 2011 | Y: P1.5-1,10-1: F4-1: ST-10138 (cc23) | Cluster 3 |
| 20310 | UK [Northern Ireland] | 2011 | Y: P1.21,10-46: F3-7: ST-9893 (cc174) |  |
| 20562 | Canada | 2002 | Y: P1.5-1,10-4: F3-4: ST-3923 (cc167) |  |
| 20563 | Canada | 2006 | Y: P1.5-1,10-4: F3-4: ST-3980 (cc167) |  |
| 21108 | UK [England] | 2011 | Y: P1.5-2,10-1: F4-1: ST-8096 (cc23) | Cluster 3 |
| 21178 | UK [England] | 2011 | Y: P1.5-1,2-2: F5-8: ST-8411 (cc23) | Cluster 2 |
| 25822 | South Africa | 2004 | Y: P1.5-1,2-2: F5-8: ST-175 (cc175) |  |
| 26066 | Sweden | 1995 | Y: P1.5-1,2-2: F-ND: ST-4988 (cc23) | Cluster 2 |
| 26090 | Sweden | 1999 | Y: P1.5-1,2-2: F5-8: ST-1625 (cc23) | Cluster 2 |
| 26115 | Sweden | 2004 | Y: P1.5-1,2-2: F5-8: ST-3801 (cc23) | Cluster 2 |
| 26140 | Sweden | 2008 | Y: P1.5-1,10-4: F4-1: ST-10302 () |  |
| 26211 | Sweden | 2011 | Y: P1.5-2,10-1: F5-12: ST-9299 (cc23) | Cluster 3 |
| 26233 | Sweden | 2012 | Y: P1.5-2,10-28: F4-1: ST-10213 (cc23) | Cluster 3 |
| 26249 | Sweden | 2012 | Y: P1.5-1,2-2: F1-18: ST-10210 (cc23) | Cluster 2 |
| 26250 | Sweden | 2012 | Y: P1.5-1,10-4: F3-4: ST-6464 () |  |
| 26611 | China | 1967 | Y: P1.5-1,2-2: F5-8: ST-175 (cc175) |  |
| 26712 | USA | 1999 | Y: P1.5-2,10-1: F4-1: ST-1621 (cc23) | Cluster 3 |
| 27497 | UK [England] | 2000 | Y: P1.21,16: F2-16: ST-10585 (cc174) |  |
| 27510 | UK [England] | 2009 | Y: P1.18-1,34: F1-5: ST-6058 (cc41/44) |  |
| 27525 | UK [England] | 2009 | Y: P1.19-3,15: F5-8: ST-5065 (cc212) |  |
| 27572 | UK [England] | 2009 | Y: P1.21,16: F3-7: ST-7850 (cc174) |  |
| 27574 | UK [England] | 2009 | Y: P1.5-1,10-4: F5-5: ST-279 (cc167) |  |
| 28183 | UK [England] | 2012 | Y: P1.5-1,10-10: F3-6: ST-10727 (cc167) |  |
| 28189 | UK [England] | 2012 | Y: P1.5-1,10-1: F4-1: ST-10729 (cc167) |  |
| 28233 | UK [England] | 2013 | Y: P1.7-2,13-1: F4-1: ST-4446 (cc23) | Cluster 3 |
| 29159 | The Netherlands | 2012 | Y: P1.5-1,10-4: F4-3: ST-784 (cc92) |  |
| 29200 | Finland | 2012 | Y: P1.5-1,10-7: F1-15: ST-884 (cc167) |  |
| 29227 | Norway | 2012 | Y: P1.5-1,2-2: F5-8: ST-8043 (cc23) | Cluster 2 |
| 29691 | UK | 2002 | Y: P1.5-1,2-2: F5-1: ST-13393 (cc22) |  |
| 30102 | Algeria | 1996 | Y: P1.5-1,10-4: F1-1: ST-167 (cc167) |  |
| 30271 | Unknown |  | Y: P1.5,2: F1-1: ST-11149 (cc11) |  |
| 30391 | The Netherlands | 2011 | Y: P1.21,4: F1-5: ST-2196 () |  |
| 30546 | Germany | 2011 | Y: P1.18-1,3: F5-8: ST-7952 () |  |
| 30594 | Germany | 2011 | Y: P1.5-1,10-1: F1-3: ST-9376 (cc167) |  |
| 30884 | Germany | 2012 | Y: P1.18-1,3: F4-3: ST-184 (cc22) |  |
| 34632 | USA | 1993 | Y: P1.5-1,2-2: F5-8: ST-1622 (cc23) | Cluster 2 |
| 34970 | Cyprus | 2012 | Y: P1.5-1,10-1: F4-1: ST-3228 (cc23) | Cluster 3 |
| 35026 | Burkina Faso | 2011 | Y: P1.21-15,16: F1-7: ST-9367 (cc10217) |  |
| 35182 | Slovenia | 2013 | Y: P1.18-1,3: F5-8: ST-11289 () |  |
| 35183 | Slovenia | 2014 | Y: P1.21,16: F3-7: ST-11206 (cc174) |  |
| 35184 | Slovenia | 2014 | Y: P1.7-2,4-18: F5-8: ST-11290 () |  |
| 35185 | Slovenia | 2014 | Y: P1.18-1,3: F3-4: ST-11287 () |  |
| 35187 | Slovenia | 2014 | Y: P1.7,30-3: F1-2: ST-53 (cc53) |  |
| 35266 | UK [Scotland] | 2009 | Y: P1.21,16: F3-7: ST-7867 (cc174) |  |
| 35523 | UK [England] | 2013 | Y: P1.5-1,10-8: F3-6: ST-11254 (cc167) |  |
| 35717 | UK [England] | 2014 | Y: P1.5-1,2-59: F1-5: ST-910 () |  |
| 35721 | UK [England] | 2014 | Y: P1.5-1,10-1: F4-1: ST-11331 (cc23) | Cluster 3 |
| 35971 | UK |  | Y: P1.21-2,28: F5-2: ST-2380 (cc35) |  |
| 35997 | UK |  | Y: P1.5,2: F4-1: ST-10683 () |  |
| 35998 | UK |  | Y: P1.5-1,10-4: F4-1: ST-11323 (cc23) | Cluster 3 |
| 36019 | UK |  | Y: P1.5-1,2-2: F5-1: ST-3651 (cc22) |  |
| 36025 | UK |  | Y: P1.5-1,10-4: F3-4: ST-10728 (cc167) |  |
| 36039 | UK |  | Y: P1.7-2,4: F1-5: ST-11324 (cc41/44) |  |
| 36046 | UK |  | Y: P1.5-1,10-1: F4-1: ST-11325 (cc23) | Cluster 3 |
| 36080 | UK |  | Y: P1.5-2,10-2: F4-1: ST-894 (cc23) | Cluster 1 |
| 36089 | UK |  | Y: P1.5,2: F4-1: ST-11326 () |  |
| 36090 | UK |  | Y: P1.5-1,10-10: F3-9: ST-13 (cc269) |  |
| 36098 | UK |  | Y: P1.5-2,10-2: F-ND: ST-183 (cc23) | Cluster 1 |
| 36112 | UK |  | Y: P1.18-1,3: F4-1: ST-1617 (cc22) |  |
| 36116 | UK |  | Y: P1.7,30-3: F1-2: ST-11327 (cc22) |  |
| 36127 | Ireland | 2015 | Y: P1.5-1,10-4: F1-7: ST-168 (cc167) |  |
| 36316 | Sweden | 2014 | Y: P1.5-2,10-1: F4-1: ST-11293 (cc23) | Cluster 3 |
| 36511 | UK |  | Y: P1.5-2,10-2: F-ND: ST-11360 (cc23) | Cluster 1 |
| 36569 | UK |  | Y: P1.5-1,10-4: F4-1: ST-11362 (cc23) | Cluster 3 |
| 36596 | UK |  | Y: P1.5-1,10-1: F4-1: ST-11398 (cc23) | Cluster 3 |
| 36614 | UK |  | Y: P1.21,16: F1-1: ST-11399 (cc167) |  |
| 36626 | UK |  | Y: P1.5-1,10-1: F-ND: ST-6525 () |  |
| 36629 | UK |  | Y: P1.18-1,3-8: F4-1: ST-1224 (cc22) |  |
| 36647 | UK |  | Y: P1.5-1,10-1: F4-1: ST-3668 (cc23) | Cluster 3 |
| 37689 | UK [England] | 2014 | Y: P1.5-1,10-1: F4-1: ST-11866 (cc23) | Cluster 3 |
| 37768 | UK [England] | 2014 | Y: P1.5-1,10-4: F4-1: ST-11472 (cc23) | Cluster 3 |
| 37808 | UK [England] | 2015 | Y: P1.5-1,10-4: F3-4: ST-11474 (cc167) |  |
| 38053 | UK [England] | 2015 | Y: P1.5-1,10-4: F4-1: ST-11875 (cc23) | Cluster 3 |
| 38087 | UK [England] | 2015 | Y: P1.5-1,2-2: F5-8: ST-11490 (cc23) | Cluster 2 |
| 38425 | UK |  | Y: P1.22,14-15: F1-2: ST-1157 (cc1157) |  |
| 38450 | UK |  | Y: P1.5-1,10-1: F4-1: ST-11361 (cc23) | Cluster 3 |
| 38983 | France |  | ND: P1.18-1,3: F1-5: ST-22 (cc22) |  |
| 39054 | UK | 2013 | Y: P1.21-2,28: F4-1: ST-11742 () |  |
| 40206 | France | 2015 | Y: P1.5-1,2-2: F5-8: ST-3582 (cc23) | Cluster 2 |
| 40249 | France | 2015 | Y: P1.21,16: F3-7: ST-1466 (cc174) |  |
| 40273 | France | 2015 | Y: P1.5-1,10-8: F3-6: ST-1627 (cc167) |  |
| 40343 | Italy | 2014 | Y: P1.5-2,10-1: F4-1: ST-11850 (cc23) | Cluster 3 |
| 40400 | France | 2015 | Y: P1.5-1,10-4: F3-6: ST-11881 (cc167) |  |
| 40474 | France | 2015 | Y: P1.5-2,10-2: F4-1: ST-11845 (cc23) | Cluster 1 |
| 40477 | France | 2015 | Y: P1.5-1,10-4: F3-4: ST-11846 (cc103) |  |
| 41225 | France | 2016 | Y: P1.18-1,3: F3-4: ST-11914 () |  |
| 41311 | Sweden | 2013 | Y: P1.5-2,10-1: F4-1: ST-11897 (cc23) | Cluster 3 |
| 41628 | Greece | 2015 | Y: P1.5-1,10-1: F4-1: ST-1655 (cc23) | Cluster 3 |
| 41748 | France | 2015 | Y: P1.5-1,10-46: F5-36: ST-103 (cc103) |  |
| 41754 | France | 2015 | Y: P1.5-2,10-2: F4-1: ST-3030 (cc23) | Cluster 1 |
| 41755 | France | 2015 | Y: P1.5-1,10-1: F3-6: ST-11927 (cc167) |  |
| 41760 | France | 2015 | Y: P1.5-1,10-46: F3-9: ST-11928 (cc103) |  |
| 41767 | France | 2015 | Y: P1.5-1,10-40: F3-6: ST-3938 (cc167) |  |
| 41770 | France | 2015 | Y: P1.5,2: F1-5: ST-11929 (cc103) |  |
| 41802 | France | 2016 | Y: P1.7-1,1: F1-6: ST-865 (cc865) |  |
| 42045 | France | 2016 | Y: P1.5,2: F3-4: ST-12009 (cc22) |  |
| 42127 | Morocco | 2013 | Y: P1.5-1,10-4: F3-6: ST-1627 (cc167) |  |
| 42200 | France | 2016 | Y: P1.5-1,10-4: F3-4: ST-13300 (cc167) |  |
| 42367 | Sweden | 2014 | Y: P1.5-2,10-1: F4-1: ST-6586 (cc23) | Cluster 3 |
| 42394 | Sweden | 2015 | Y: P1.5-3,10-1: F3-6: ST-12014 () |  |
| 42429 | Sweden | 2016 | Y: P1.5-2,10-1: F4-1: ST-12015 (cc23) | Cluster 3 |
| 42685 | Ethiopia | 2014 | Y: P1.5-1,10-8: F1-3: ST-2880 (cc167) |  |
| 43539 | South Africa | 2016 | Y: P1.5-1,10-8: F1-3: ST-767 (cc167) |  |
| 44189 | UK [England] | 2015 | Y: P1.5-1,10-10: F4-1: ST-12353 (cc23) | Cluster 3 |
| 44190 | UK [England] | 2015 | Y: P1.5-1,10-10: F4-1: ST-9453 (cc23) | Cluster 3 |
| 44363 | UK [England] | 2015 | Y: P1.17-1,10-1: F5-25: ST-12230 () |  |
| 44427 | UK [England] | 2015 | Y: P1.5-1,10-1: F4-1: ST-12176 (cc23) | Cluster 3 |
| 44507 | UK [England] | 2015 | Y: P1.5-1,10-1: F4-1: ST-9842 (cc23) | Cluster 3 |
| 45364 | The Netherlands | 2016 | Y: P1.18-1,3: F3-4: ST-12348 () |  |
| 46373 | Sweden | 2016 | Y: P1.5-1,10-6: F5-1: ST-114 (cc22) |  |
| 46538 | Tunisia | 2015 | Y: P1.5-1,2-2: F5-8: ST-13354 (cc23) | Cluster 2 |
| 46615 | UK [Scotland] | 2014 | Y: P1.5-1,10-4: F5-5: ST-766 (cc167) |  |
| 46696 | UK [Scotland] | 2015 | Y: P1.5-1,10-10: F4-1: ST-11280 (cc23) | Cluster 3 |
| 47109 | Italy | 2016 | Y: P1.5-2,10-2: F2-13: ST-9624 (cc23) | Cluster 1 |
| 47202 | Sweden |  | ND: P1.21,26: F5-2: ST-913 (cc60) |  |
| 49657 | UK [Wales] | 2015 | Y: P1.5-1,10-4: F3-4: ST-12641 (cc167) |  |
| 49708 | UK [England] | 2015 | Y: P1.5-1,10-8: F3-6: ST-12651 (cc167) |  |
| 49730 | UK [England] | 2015 | Y: P1.5-1,10-4: F4-1: ST-10294 (cc23) | Cluster 3 |
| 49776 | UK [England] | 2015 | Y: P1.5-1,10-4: F5-8: ST-1760 (cc167) |  |
| 49780 | UK [England] | 2015 | Y: P1.5-1,10-10: F4-1: ST-12666 (cc23) | Cluster 3 |
| 49802 | UK [England] | 2015 | Y: P1.5-1,10-1: F4-1: ST-11754 (cc23) | Cluster 3 |
| 49852 | UK [England] | 2015 | Y: P1.5-2,10-1: F4-1: ST-12578 (cc23) | Cluster 3 |
| 49890 | UK [England] | 2015 | Y: P1.5-1,10-4: F4-1: ST-6463 (cc23) | Cluster 3 |
| 49937 | UK [England] | 2015 | Y: P1.5-1,10-1: F4-1: ST-12677 (cc23) | Cluster 3 |
| 49947 | UK [England] | 2015 | Y: P1.5-1,10-1: F4-1: ST-10458 (cc23) | Cluster 3 |
| 50000 | UK [England] | 2015 | Y: P1.5-1,10-4: F3-4: ST-10730 (cc167) |  |
| 50239 | UK [England] | 2015 | Y: P1.5-2,10-1: F4-1: ST-12231 (cc23) | Cluster 3 |
| 50361 | UK [England] | 2015 | Y: P1.19,15-41: F5-1: ST-34 (cc32) |  |
| 50374 | UK [England] | 2015 | Y: P1.5-1,10-4: F4-1: ST-12703 (cc23) | Cluster 3 |
| 51009 | France | 2016 | Y: P1.5-2,10-1: F4-1: ST-13356 (cc23) | Cluster 3 |
| 51041 | Finland | 2016 | Y: P1.18-1,3: F3-4: ST-5436 () |  |
| 51209 | France | 2017 | Y: P1.5-2,10-1: F4-1: ST-10732 (cc23) | Cluster 3 |
| 51214 | Finland | 2017 | Y: P1.5-2,10-1: F4-1: ST-2692 (cc23) | Cluster 3 |
| 51290 | UK [England] | 2016 | Y: P1.18-7,9: F3-9: ST-13221 (cc103) |  |
| 52429 | France | 2017 | Y: P1.5-2,10-2: F5-18: ST-13358 (cc23) | Cluster 1 |
| 52511 | China | 1967 | Y: P1.5-1,10-4: F5-8: ST-10368 (cc175) |  |
| 52596 | UK [England] | 2015 | Y: P1.18-1,3: F3-9: ST-1418 (cc103) |  |
| 52626 | UK [Scotland] | 2015 | Y: P1.5-1,10-1: F4-1: ST-12414 (cc23) | Cluster 3 |
| 52784 | France | 2017 | Y: P1.5-2,10-1: F5-12: ST-13363 (cc23) | Cluster 3 |
| 52905 | UK [Scotland] | 1989 | Y: P1.5-1,10-4: F5-8: ST-12938 (cc167) |  |
| 52936 | Italy | 2016 | Y: P1.5-1,2-2: F4-1: ST-3587 (cc23) | Cluster 1 |
| 53085 | UK [England] | 2016 | Y: P1.5-1,10-1: F4-1: ST-13092 (cc23) | Cluster 3 |
| 53106 | UK [England] | 2016 | Y: P1.5-1,10-4: F4-1: ST-13093 (cc23) | Cluster 3 |
| 53251 | UK [England] | 2017 | Y: P1.5-1,10-1: F5-12: ST-11520 () |  |
| 53720 | Italy |  | Y: P1.5-2,10-2: F2-13: ST-13360 (cc23) | Cluster 1 |
| 53969 | UK [England] | 2008 | Y: P1.21,16: F3-7: ST-8510 (cc174) |  |
| 54033 | Brazil | 2014 | Y: P1.18-1,3: F3-4: ST-3015 () |  |
| 54058 | Brazil | 2014 | ND: P1.5-1,10-3: F1-7: ST-5770 (cc175) |  |
| 54484 | Sweden | 2017 | Y: P1.5-1,10-4: F3-6: ST-13101 (cc167) |  |
| 54488 | Sweden | 2017 | Y: P1.5-2,10-1: F4-1: ST-13108 (cc23) | Cluster 3 |
| 54593 | France | 2002 | Y: P1.5-2,10-2: F4-1: ST-13355 (cc23) | Cluster 1 |
| 55539 | Ireland | 2016 | W: P1.5-2,10-1: F5-8: ST-9316 () |  |
| 55568 | Ireland | 2017 | W: P1.5,44-1: F1-1: ST-10651 (cc11) |  |
| 56453 | France | 2017 | Y: P1.5-1,10-1: F4-1: ST-1378 (cc23) | Cluster 3 |
| 56639 | Canada | 2017 | Y: P1.5,2: F5-8: ST-11 (cc11) |  |
| 56659 | Canada | 2016 | Y: P1.5-1,2-2: F5-8: ST-12163 (cc23) | Cluster 2 |
| 57401 | Ireland | 2017 | Y: P1.5-1,10-4: F5-12: ST-5987 (cc103) |  |
| 57438 | Ireland | 2017 | Y: P1.5-1,10-1: F4-1: ST-13170 (cc23) | Cluster 3 |
| 57469 | Ireland | 2017 | Y: P1.5-1,10-4: F3-4: ST-9579 (cc167) |  |
| 57498 | Ireland | 2017 | ND: P1.18-1,3: F4-1: ST-1281 (cc22) |  |
| 57516 | Ireland | 2017 | Y: P1.5-1,10-22: F4-1: ST-11388 (cc23) | Cluster 3 |
| 58163 | Sweden | 2017 | Y: P1.5-2,10-1: F4-1: ST-4183 (cc23) | Cluster 3 |
| 58345 | Burkina Faso | 2012 | Y: P1.5-1,2-2: F5-8: ST-4375 (cc23) | Cluster 2 |
| 59124 | USA | 2006 | Y: P1.5-1,10-62: F4-1: ST-5918 (cc167) |  |
| 59333 | USA | 2007 | Y: P1.5-1,10-4: F3-4: ST-11176 (cc167) |  |
| 59452 | USA | 2008 | Y: P1.5-1,2-2: F5-8: ST-893 (cc23) | Cluster 2 |
| 59559 | Australia | 2017 | Y: P1.5,2: F4-1: ST-13604 (cc23) | Cluster 3 |
| 59696 | South Africa | 2016 | Y: P1.5-2,10-1: F4-1: ST-13846 (cc23) | Cluster 3 |
| 59828 | South Africa | 2017 | Y: P1.5-1,2-2: F1-47: ST-6218 (cc175) |  |
| 59829 | South Africa | 2017 | Y: P1.5-2,10-1: F4-1: ST-4245 (cc23) | Cluster 3 |
| 59873 | South Africa | 2017 | Y: P1.18,25-44: F5-5: ST-823 (cc198) |  |
| 59891 | South Africa | 2017 | Y: P1.5-1,10-7: F1-15: ST-13848 (cc167) |  |
| 59938 | South Africa | 2017 | Y: P1.5-1,10-1: F1-7: ST-2076 (cc167) |  |
| 60008 | Australia | 2011 | Y: P1.5-1,2-2: F5-8: ST-6799 (cc23) | Cluster 2 |
| 60009 | Australia | 2011 | Y: P1.5-2,10-1: F4-1: ST-23 (cc23) | Cluster 3 |
| 60020 | Australia | 2017 | Y: P1.5-1,2-2: F5-8: ST-6800 (cc23) | Cluster 2 |
| 61355 | Australia |  | Y: P1.18-37,25: F5-70: ST-13767 (cc41/44) |  |
| 83759 | Sweden |  | Y: P1.5-2,10-1: F5-12: ST-9083 (cc23) | Cluster 3 |
| 84042 | Italy |  | Y: P1.5-2,10-2: F2-13: ST-9253 (cc23) | Cluster 1 |
| 84077 | Italy |  | Y: P1.21,4: F4-1: ST-1768 () |  |

**Supplementary Table 2** List of cc23:Y isolates used in further analyses.

| **PubMLST id** | **isolate** | **country** | **year** | **strain_designation** | **Age range if known/available** | **ClonalFrameML and TreeWAS analyses** |
| --- | --- | --- | --- | --- | --- | --- |
| 19962 | M10 240503 | UK [England] | 2010 | Y: P1.5-1,2-2: F1-96: ST-23 (cc23) | <65 | 3 |
| 19963 | M10 240505 | UK [England] | 2010 | Y: P1.5-1,10-10: F4-1: ST-1655 (cc23) | <65 | 3 |
| 19964 | M10 240507 | UK [England] | 2010 | Y: P1.21,16-5: &Delta;: ST-183 (cc23) | >=65 | 1, 2 |
| 19970 | M10 240520 | UK [England] | 2010 | Y: P1.5-1,10-1: F4-1: ST-1655 (cc23) | <65 | 1, 2 |
| 19976 | M10 240530 | UK [England] | 2010 | Y: P1.5-2,10-2: F3-1: ST-9813 (cc23) | >=65 | 3 |
| 19977 | M10 240531 | UK [England] | 2010 | Y: P1.5-1,10-1: F4-1: ST-1655 (cc23) | <65 | 2 |
| 19979 | M10 240534 | UK [England] | 2010 | Y: P1.5-1,10-1: F4-1: ST-1655 (cc23) | <65 | 1 |
| 19980 | M10 240536 | UK [England] | 2010 | Y: P1.5-1,10-1: F4-1: ST-1655 (cc23) | <65 | 1, 3 |
| 19981 | M10 240540 | UK [England] | 2010 | Y: P1.5-1,10-10: F4-1: ST-9814 (cc23) | >=65 | 2 |
| 19998 | M10 240580 | UK [England] | 2010 | Y: P1.5-1,10-1: F4-1: ST-1655 (cc23) | <65 | 2, 3 |
| 20006 | M10 240590 | UK [England] | 2010 | Y: P1.5-1,10-4: F4-1: ST-23 (cc23) | >=65 | 2, 3 |
| 20032 | M10 240632 | UK [England] | 2010 | Y: P1.5-1,10-1: F4-1: ST-1655 (cc23) | >=65 | 1 |
| 20033 | M10 240633 | UK [England] | 2010 | Y: P1.5-1,10-10: F4-1: ST-1655 (cc23) | <65 | 2 |
| 20041 | M10 240643 | UK [England] | 2010 | Y: P1.5-2,10-1: F4-1: ST-23 (cc23) | >=65 | 1, 3 |
| 20067 | M10 240685 | UK [England] | 2010 | Y: P1.5-1,10-1: F4-1: ST-1655 (cc23) | <65 | 1, 2 |
| 20074 | M10 240700 | UK [England] | 2010 | Y: P1.5-1,10-4: F4-1: ST-6463 (cc23) | >=65 | 1, 2 |
| 20099 | M10 240732 | UK [England] | 2010 | Y: P1.5-1,2-2: F5-8: ST-23 (cc23) | >=65 | 1, 2, 3 |
| 20104 | M10 240745 | UK [England] | 2010 | Y: P1.5-1,10-4: F4-1: ST-1655 (cc23) | <65 | 2, 3 |
| 20128 | M10 240776 | UK [England] | 2010 | Y: P1.5-1,10-4: F4-1: ST-1655 (cc23) | <65 | 1, 2, 3 |
| 20129 | M10 240777 | UK [England] | 2010 | Y: P1.5-1,2-2: F5-8: ST-23 (cc23) | <65 | 1, 3 |
| 20135 | M10 240786 | UK [England] | 2010 | Y: P1.5-1,10-1: F4-1: ST-9831 (cc23) | <65 | 2, 3 |
| 20136 | M10 240787 | UK [England] | 2010 | Y: P1.5-1,10-10: F4-1: ST-1655 (cc23) | <65 | 2, |
| 20143 | M10 240798 | UK [England] | 2010 | Y: P1.5-1,10-1: F4-1: ST-1655 (cc23) | >=65 | 1, 2, 3 |
| 20146 | M10 240804 | UK [England] | 2010 | Y: P1.5-1,10-1: F4-1: ST-1655 (cc23) | <65 | 3 |
| 20164 | M11 240000 | UK [England] | 2011 | Y: P1.5-1,10-4: F4-1: ST-23 (cc23) | >=65 | 1, 2, 3 |
| 20169 | M11 240005 | UK [England] | 2011 | Y: P1.5-1,2-2: F5-8: ST-23 (cc23) | >=65 | 1 |
| 20171 | M11 240007 | UK [England] | 2011 | Y: P1.5-1,10-10: F4-1: ST-1655 (cc23) | <65 | 1, 2, 3 |
| 20193 | M11 240031 | UK [England] | 2011 | Y: P1.5-1,10-1: F4-1: ST-1655 (cc23) | >=65 | 1, 2 |
| 20197 | M11 240036 | UK [Wales] | 2011 | Y: P1.7-2,10-1: F4-1: ST-1655 (cc23) | >=65 | 2 |
| 20204 | M11 240043 | UK [England] | 2011 | Y: P1.5-1,10-1: F4-1: ST-9842 (cc23) | >=65 | 2, 3 |
| 20224 | M11 240065 | UK [England] | 2011 | Y: P1.5-1,10-1: F4-1: ST-1655 (cc23) | <65 | 1, 2, 3 |
| 20245 | M11 240096 | UK [England] | 2011 | Y: P1.5-1,10-1: F4-1: ST-1655 (cc23) | >=65 | 1, 3 |
| 20256 | M11 240116 | UK [England] | 2011 | Y: P1.5-1,10-1: F4-1: ST-1655 (cc23) | <65 | 1, 2 |
| 20264 | M11 240126 | UK [England] | 2011 | Y: P1.5-1,10-4: F4-1: ST-23 (cc23) | <65 | 1, 2 |
| 20281 | M11 240157 | UK [England] | 2011 | Y: P1.5-1,10-1: F4-1: ST-10138 (cc23) | <65 | 1 |
| 20286 | M11 240166 | UK [England] | 2011 | Y: P1.5-2,10-1: F4-1: ST-23 (cc23) | <65 | 1, 2, 3 |
| 20302 | M11 240192 | UK [Wales] | 2011 | Y: P1.5-1,10-1: F4-1: ST-1655 (cc23) | <65 | 2 |
| 20309 | M11 240210 | UK [England] | 2011 | Y: P1.5-1,2-2: F5-8: ST-23 (cc23) | >=65 | 1 |
| 20314 | M11 240215 | UK [England] | 2011 | Y: P1.5-1,10-1: F4-1: ST-1655 (cc23) | <65 | 2 |
| 20315 | M11 240216 | UK [England] | 2011 | Y: P1.5-1,10-1: F4-1: ST-1655 (cc23) | <65 | 2 |
| 20317 | M11 240227 | UK [England] | 2011 | Y: P1.5-1,10-4: F4-1: ST-6463 (cc23) | >=65 | 1, 3 |
| 20335 | M11 240258 | UK [England] | 2011 | Y: P1.5-1,2-2: F5-8: ST-23 (cc23) | >=65 | 1, 3 |
| 20346 | M11 240277 | UK [England] | 2011 | Y: P1.5-1,10-1: F4-1: ST-1655 (cc23) | <65 | 2, 3 |
| 20347 | M11 240278 | UK [England] | 2011 | Y: P1.7-2,30-4: F4-1: ST-1655 (cc23) | <65 | 3 |
| 20351 | M11 240283 | UK [England] | 2011 | Y: P1.5-2,10-1: F4-1: ST-23 (cc23) | <65 | 1,2 ,3 |
| 20363 | M11 240298 | UK [England] | 2011 | Y: P1.5-1,10-1: F4-1: ST-1655 (cc23) | >=65 | 2 |
| 20372 | M11 240312 | UK [England] | 2011 | Y: P1.5-1,10-4: F4-1: ST-23 (cc23) | <65 | 1 |
| 20373 | M11 240313 | UK [England] | 2011 | Y: P1.5-1,10-1: F4-1: ST-1655 (cc23) | <65 | 1,2 |
| 20379 | M11 240319 | UK [England] | 2011 | Y: P1.5-1,10-1: F4-1: ST-1655 (cc23) | <65 | 1 |
| 20380 | M11 240322 | UK [England] | 2011 | Y: P1.5-1,10-1: F4-1: ST-1655 (cc23) | <65 | 1, 3 |
| 20383 | M11 240326 | UK [England] | 2011 | Y: P1.5-1,10-1: F4-1: ST-1655 (cc23) | >=65 | 1, 2 |
| 20404 | M11 240358 | UK [England] | 2011 | W/Y: P1.5-1,2-2: F5-8: ST-23 (cc23) | <65 | 1, 2, 3 |
| 20414 | M11 240372 | UK [England] | 2011 | Y: P1.5-1,10-4: F4-1: ST-1655 (cc23) | <65 | 1, 3 |
| 20430 | M11 240396 | UK [England] | 2011 | Y: P1.5-1,10-1: F4-1: ST-1655 (cc23) | >=65 | 1 |
| 20433 | M11 240399 | UK [England] | 2011 | Y: P1.5-1,10-1: F4-1: ST-1655 (cc23) | <65 | 1, 2 |
| 20440 | M11 240411 | UK [England] | 2011 | Y: P1.5-1,2-2: F5-8: ST-4183 (cc23) | <65 | 1, 2 |
| 20451 | M11 240430 | UK [England] | 2011 | Y: P1.5-2,10-1: F4-1: ST-23 (cc23) | <65 | 3 |
| 20454 | M11 240435 | UK [England] | 2011 | Y: P1.5-1,10-1: F4-1: ST-1655 (cc23) | <65 | 1, 2, 3 |
| 20459 | M11 240442 | UK [England] | 2011 | Y: P1.5-1,10-1: F4-1: ST-23 (cc23) | >=65 | 1, 3 |
| 21094 | M11 240446 | UK [England] | 2011 | Y: P1.5-1,10-1: F4-1: ST-1655 (cc23) | >=65 | 1, 2 |
| 21105 | M11 240461 | UK [England] | 2011 | Y: P1.5-1,2-2: F5-8: ST-23 (cc23) | <65 | 1 |
| 21107 | M11 240465 | UK [England] | 2011 | Y: P1.5-1,10-4: F4-1: ST-1655 (cc23) | >=65 | 3 |
| 21108 | M11 240466 | UK [England] | 2011 | Y: P1.5-2,10-1: F4-1: ST-8096 (cc23) | <65 | 1 |
| 21130 | M11 240494 | UK [England] | 2011 | Y: P1.5-1,10-1: F4-1: ST-1655 (cc23) | >=65 | 2 |
| 21138 | M11 240507 | UK [England] | 2011 | Y: P1.5-1,10-1: F4-1: ST-1655 (cc23) | >=65 | 1, 2, 3 |
| 21148 | M11 240599 | UK [England] | 2011 | Y: P1.5-1,10-1: F4-1: ST-1655 (cc23) | >=65 | 1, 2, 3 |
| 21151 | M11 240657 | UK [England] | 2011 | Y: P1.5-1,10-4: F4-1: ST-23 (cc23) | >=65 | 2 |
| 21153 | M11 240710 | UK [England] | 2011 | Y: P1.5-1,10-1: F4-1: ST-1655 (cc23) | >=65 | 3 |
| 21157 | M11 240717 | UK [Northern Ireland] | 2011 | Y: P1.5-1,10-1: F4-1: ST-1655 (cc23) | >=65 | 2, 3 |
| 21158 | M11 240719 | UK [England] | 2011 | Y: P1.5-1,2-2: F5-8: ST-3582 (cc23) | <65 | 2, 3 |
| 21167 | M11 240731 | UK [England] | 2011 | Y: P1.5-1,10-1: F4-1: ST-1655 (cc23) | >=65 | 1, 2 |
| 21169 | M11 240734 | UK [England] | 2011 | Y: P1.5-1,2-2: F5-8: ST-23 (cc23) | <65 | 3 |
| 21172 | M11 240737 | UK [Wales] | 2011 | Y: P1.5-1,10-1: F4-1: ST-1655 (cc23) | >=65 | 2 |
| 21178 | M11 240745 | UK [England] | 2011 | Y: P1.5-1,2-2: F5-8: ST-8411 (cc23) | <65 | 2 |
| 21183 | M11 240765 | UK [England] | 2011 | Y: P1.5-1,10-1: F4-1: ST-1655 (cc23) | <65 | 1, 2, 3 |
| 21187 | M11 240772 | UK [England] | 2011 | Y: P1.5-1,10-4: F4-1: ST-1655 (cc23) | >=65 | 2 |
| 21192 | M11 240779 | UK [England] | 2011 | Y: P1.5-1,10-4: F4-1: ST-6463 (cc23) | >=65 | 1, 2 |
| 21198 | M11 240788 | UK [England] | 2011 | Y: P1.5-1,10-4: F4-1: ST-1655 (cc23) | <65 | 2 |
| 21201 | M11 240793 | UK [England] | 2011 | Y: P1.5-1,2-2: F5-8: ST-23 (cc23) | >=65 | 1, 3 |
| 21205 | M11 240801 | UK [England] | 2011 | Y: P1.5-2,10-12: F4-1: ST-23 (cc23) | <65 | 1 |
| 21223 | M11 240982 | UK [Northern Ireland] | 2011 | Y: P1.5-1,10-1: F4-1: ST-1655 (cc23) | <65 | 2, 3 |
| 21227 | M11 240987 | UK [England] | 2011 | Y: P1.5-1,2-2: F1-96: ST-23 (cc23) | >=65 | 1, 2, 3 |
| 21229 | M11 240991 | UK [England] | 2011 | Y: P1.5-1,10-1: F4-1: ST-1655 (cc23) | <65 | 1, 3 |
| 21250 | M11 241035 | UK [England] | 2011 | Y: P1.5-1,10-1: F4-1: ST-1655 (cc23) | <65 | 2 |
| 21255 | M11 241042 | UK [England] | 2011 | Y: P1.5-1,10-1: F4-1: ST-1655 (cc23) | <65 | 3 |
| 21259 | M11 241047 | UK [England] | 2011 | Y: P1.5-1,10-4: F4-1: ST-1655 (cc23) | <65 | 1, 2 |
| 21262 | M11 241051 | UK [England] | 2011 | Y: P1.5-1,2-2: F5-8: ST-23 (cc23) | <65 | 3 |
| 21272 | M11 241065 | UK [England] | 2011 | Y: P1.5-1,10-1: F4-1: ST-1655 (cc23) | <65 | 1, 3 |
| 21274 | M11 241067 | UK [England] | 2011 | Y: P1.5-1,10-1: F4-1: ST-1655 (cc23) | <65 | 1, 2 |
| 21276 | M11 241069 | UK [England] | 2011 | Y: P1.5-1,2-2: F5-8: ST-23 (cc23) | <65 | 2 |
| 21283 | M11 241078 | UK [England] | 2011 | Y: P1.5-1,10-10: F4-1: ST-1655 (cc23) | >=65 | 2 |
| 21293 | M12 240011 | UK [England] | 2012 | Y: P1.5-1,2-2: F5-8: ST-23 (cc23) | <65 | 1, 2, 3 |
| 21294 | M12 240012 | UK [England] | 2012 | Y: P1.5-1,10-1: F4-1: ST-1655 (cc23) | >=65 | 1, 2 |
| 21295 | M12 240013 | UK [England] | 2012 | Y: P1.5-1,10-1: F4-1: ST-1655 (cc23) | <65 | 3 |
| 21304 | M12 240024 | UK [England] | 2012 | Y: P1.5-1,10-1: F4-1: ST-1655 (cc23) | >=65 | 2 |
| 21338 | M12 240072 | UK [England] | 2012 | Y: P1.5-1,10-1: F4-1: ST-1655 (cc23) | >=65 | 1, 3 |
| 21343 | M12 240080 | UK [England] | 2012 | Y: P1.5-1,10-4: F4-1: ST-1655 (cc23) | <65 | 1 |
| 21346 | M12 240084 | UK [England] | 2012 | Y: P1.5-1,10-10: F4-1: ST-1655 (cc23) | >=65 | 1, 2 |
| 21356 | M12 240098 | UK [England] | 2012 | Y: P1.5-1,10-1: F4-1: ST-1655 (cc23) | <65 | 2 |
| 21366 | M12 240115 | UK [England] | 2012 | Y: P1.5-1,10-1: F4-1: ST-1655 (cc23) | <65 | 2, 3 |
| 21372 | M12 240122 | UK [England] | 2012 | Y: P1.5-1,10-1: F4-1: ST-1655 (cc23) | <65 | 2 |
| 21373 | M12 240123 | UK [England] | 2012 | Y: P1.5-1,10-1: F4-1: ST-1655 (cc23) | >=65 | 1, 3 |
| 21399 | M12 240166 | UK [England] | 2012 | Y: P1.5-1,2-2: F5-8: ST-23 (cc23) | >=65 | 1, 3 |
| 21403 | M12 240171 | UK [England] | 2012 | Y: P1.5-1,10-1: F4-1: ST-1655 (cc23) | <65 | 1 |
| 21404 | M12 240174 | UK [England] | 2012 | Y: P1.5-1,2-2: F5-8: ST-23 (cc23) | >=65 | 1, 2, 3 |
| 21408 | M12 240180 | UK [England] | 2012 | Y: P1.5-1,10-10: F4-1: ST-1655 (cc23) | >=65 | 1 |
| 21410 | M12 240184 | UK [Wales] | 2012 | Y: P1.5-1,10-4: F4-1: ST-23 (cc23) | <65 | 2, 3 |
| 21440 | M12 240230 | UK [England] | 2012 | Y: P1.5-2,10-1: F4-1: ST-23 (cc23) | >=65 | 1, 2, 3 |
| 21443 | M12 240237 | UK [England] | 2012 | Y: P1.5-1,10-4: F4-1: ST-1655 (cc23) | >=65 | 1, 2, 3 |
| 21447 | M12 240241 | UK [England] | 2012 | Y: P1.5-2,10-1: F4-1: ST-23 (cc23) | <65 | 1, 2 |
| 21448 | M12 240242 | UK [England] | 2012 | Y: P1.5-1,10-1: F4-1: ST-1655 (cc23) | <65 | 2 |
| 21451 | M12 240248 | UK [England] | 2012 | Y: P1.5-1,10-4: F4-1: ST-6463 (cc23) | >=65 | 1, 2, 3 |
| 21466 | M12 240272 | UK [England] | 2012 | Y: P1.5-1,10-1: F4-1: ST-1655 (cc23) | <65 | 2 |
| 21472 | M12 240288 | UK [England] | 2012 | Y: P1.5-2,10-1: F4-1: ST-23 (cc23) | <65 | 2 |
| 21473 | M12 240289 | UK [England] | 2012 | Y: P1.5-1,10-4: F4-1: ST-10294 (cc23) | <65 | 2 |
| 21475 | M12 240291 | UK [England] | 2012 | Y: P1.5-1,10-4: F4-1: ST-23 (cc23) | >=65 | 1, 2, 3 |
| 21476 | M12 240293 | UK [England] | 2012 | Y: P1.5-1,10-1: F4-1: ST-1655 (cc23) | <65 | 2, 3 |
| 21480 | M12 240300 | UK [England] | 2012 | Y: P1.5-1,10-1: F4-1: ST-1655 (cc23) | <65 | 1, 2 |
| 21493 | M12 240318 | UK [England] | 2012 | Y: P1.5-1,10-1: F4-1: ST-1655 (cc23) | <65 | 1 |
| 21497 | M12 240322 | UK [England] | 2012 | Y: P1.5-1,2-2: F5-8: ST-23 (cc23) | >=65 | 1, 2, 3 |
| 21502 | M12 240328 | UK [England] | 2012 | Y: P1.5-1,10-4: F4-1: ST-23 (cc23) | >=65 | 2 |
| 26883 | 12023_2012 | Ireland | 2012 | Y: P1.5-1,10-1: F4-1: ST-1655 (cc23) |  |  |
| 28130 | M12 240881 | UK [England] | 2012 | W/Y: P1.5-1,10-1: F4-1: ST-1655 (cc23) | <65 | 3 |
| 28165 | M12 240342 | UK [England] | 2012 | Y: P1.5-1,10-4: F4-1: ST-23 (cc23) | >=65 | 1, 2, 3 |
| 28166 | M12 240343 | UK [Northern Ireland] | 2012 | Y: P1.5-1,10-1: F4-1: ST-1655 (cc23) | >=65 | 1, 3 |
| 28167 | M12 240643 | UK [England] | 2012 | Y: P1.5-1,10-1: F4-1: ST-1655 (cc23) | >=65 | 1, 2, |
| 28168 | M12 240646 | UK [England] | 2012 | Y: P1.5-1,10-37: F4-1: ST-1655 (cc23) | >=65 | 2 |
| 28169 | M12 240661 | UK [England] | 2012 | Y: P1.5-2,10-1: F5-12: ST-23 (cc23) | <65 | 2 |
| 28170 | M12 240701 | UK [England] | 2012 | Y: P1.5-1,10-4: F4-1: ST-23 (cc23) | <65 | 1, 2 |
| 28171 | M12 240713 | UK [Wales] | 2012 | Y: P1.5-1,10-1: F4-1: ST-1655 (cc23) | <65 | 2, 3 |
| 28172 | M12 240715 | UK [England] | 2012 | Y: P1.5-2,10-1: F4-1: ST-23 (cc23) | >=65 | 1, 3 |
| 28174 | M12 240728 | UK [England] | 2012 | Y: P1.5-1,10-4: F4-1: ST-23 (cc23) | >=65 | 1, 2,3 |
| 28175 | M12 240746 | UK [England] | 2012 | Y: P1.5-2,10-2: F4-1: ST-3030 (cc23) | <65 | 1, 2 |
| 28177 | M12 240760 | UK [England] | 2012 | Y: P1.5-1,10-1: F4-1: ST-1655 (cc23) | <65 | 3 |
| 28178 | M12 240767 | UK [England] | 2012 | Y: P1.5-1,10-10: F4-1: ST-1655 (cc23) | <65 | 1, 3 |
| 28179 | M12 240779 | UK [England] | 2012 | Y: P1.5-1,10-10: F4-1: ST-1655 (cc23) | <65 | 2, |
| 28181 | M12 240788 | UK [England] | 2012 | Y: P1.5-1,10-4: F4-1: ST-10294 (cc23) | >=65 | 2, 3 |
| 28182 | M12 240791 | UK [England] | 2012 | Y: P1.5-1,10-1: F4-1: ST-1655 (cc23) | >=65 | 3 |
| 28185 | M12 240799 | UK [England] | 2012 | Y: P1.5-1,2-2: F5-8: ST-23 (cc23) | <65 | 2, |
| 28186 | M12 240801 | UK [England] | 2012 | Y: P1.5-1,10-1: F4-1: ST-1655 (cc23) | <65 | 1, |
| 28188 | M12 240816 | UK [England] | 2012 | Y: P1.5-1,2-2: F5-8: ST-23 (cc23) | <65 | 1, 3 |
| 28190 | M12 240822 | UK [England] | 2012 | Y: P1.5-1,2-28: F5-8: ST-23 (cc23) | <65 | 3 |
| 28191 | M12 240840 | UK [England] | 2012 | Y: P1.5-1,2-2: F5-8: ST-23 (cc23) | <65 | 2, 3 |
| 28192 | M12 240857 | UK [England] | 2012 | Y: P1.5-1,10-4: F4-1: ST-1655 (cc23) | <65 | 1, 3 |
| 28193 | M12 240885 | UK [England] | 2012 | Y: P1.5-1,10-1: F4-1: ST-1655 (cc23) | >=65 | 1, 2, 3 |
| 28194 | M12 240886 | UK [England] | 2012 | Y: P1.5-1,2-2: F5-8: ST-23 (cc23) | <65 | 3 |
| 28195 | M12 240888 | UK [England] | 2012 | Y: P1.5-2,10-1: F4-1: ST-23 (cc23) | >=65 | 2, 3 |
| 28196 | M12 240894 | UK [England] | 2012 | Y: P1.5-1,10-1: F4-1: ST-1655 (cc23) | >=65 | 2, |
| 28197 | M12 240897 | UK [England] | 2012 | Y: P1.5-2,10-2: F2-13: ST-23 (cc23) | <65 | 1, 2 |
| 28198 | M13 240011 | UK [England] | 2013 | Y: P1.5-1,10-4: F4-1: ST-1655 (cc23) | >=65 | 1, 3 |
| 28200 | M13 240035 | UK [England] | 2013 | Y: P1.5-1,10-1: F4-1: ST-1655 (cc23) | >=65 | 3 |
| 28201 | M13 240044 | UK [Northern Ireland] | 2013 | Y: P1.5-1,10-1: F4-1: ST-1655 (cc23) | <65 | 3 |
| 28205 | M13 240064 | UK [England] | 2013 | Y: P1.5-1,10-12: F4-1: ST-1655 (cc23) | >=65 | 1, 2, 3 |
| 28206 | M13 240073 | UK [England] | 2013 | Y: P1.5-2,10-1: F4-1: ST-23 (cc23) | >=65 | 1, 2, 3 |
| 28207 | M13 240087 | UK [England] | 2013 | Y: P1.5-1,10-1: F4-1: ST-1655 (cc23) | >=65 | 1, 2 |
| 28208 | M13 240099 | UK [England] | 2013 | Y: P1.5-1,10-1: F4-1: ST-1655 (cc23) | <65 | 2 |
| 28209 | M13 240102 | UK [England] | 2013 | Y: P1.5-1,10-1: F4-1: ST-1655 (cc23) | <65 | 1 |
| 28210 | M13 240116 | UK [England] | 2013 | Y: P1.5-1,10-1: F4-1: ST-1655 (cc23) | <65 | 2 |
| 28212 | M13 240131 | UK [England] | 2013 | Y: P1.5-2,10-1: F4-1: ST-23 (cc23) | >=65 | 3 |
| 28214 | M13 240144 | UK [England] | 2013 | Y: P1.5-1,10-10: F4-1: ST-1655 (cc23) | >=65 | 1, 2, 3 |
| 28215 | M13 240146 | UK [England] | 2013 | Y: P1.5-1,10-1: F4-1: ST-1655 (cc23) | <65 | 1, 2, 3 |
| 28216 | M13 240154 | UK [England] | 2013 | Y: P1.5-1,10-1: F4-1: ST-1655 (cc23) | <65 | 1, 3 |
| 28217 | M13 240165 | UK [England] | 2013 | Y: P1.5-1,10-1: F4-1: ST-1655 (cc23) | >=65 | 2 |
| 28218 | M13 240184 | UK [England] | 2013 | Y: P1.5,2: F4-1: ST-1655 (cc23) | <65 | 1, 2 |
| 28219 | M13 240188 | UK [England] | 2013 | Y: P1.5-1,10-4: F4-1: ST-1655 (cc23) | <65 | 2 |
| 28220 | M13 240194 | UK [England] | 2013 | Y: P1.5-1,10-1: F4-1: ST-1655 (cc23) | <65 | 1, 2 |
| 28221 | M13 240200 | UK [England] | 2013 | Y: P1.5-1,10-1: F4-1: ST-1655 (cc23) | <65 | 2 |
| 28222 | M13 240203 | UK [England] | 2013 | Y: P1.5-2,10-1: F-ND: ST-23 (cc23) | >=65 | 1, 2 |
| 28223 | M13 240218 | UK [England] | 2013 | Y: P1.5-1,10-1: F4-1: ST-1655 (cc23) | <65 | 1 |
| 28224 | M13 240220 | UK [England] | 2013 | Y: P1.5-1,10-1: F4-1: ST-1655 (cc23) | >=65 | 2 |
| 28226 | M13 240242 | UK [England] | 2013 | Y: P1.5-2,10-2: F2-13: ST-23 (cc23) | >=65 | 1, 2 |
| 28227 | M13 240244 | UK [England] | 2013 | Y: P1.5-1,10-1: F4-1: ST-1655 (cc23) | <65 | 1 |
| 28228 | M13 240265 | UK [England] | 2013 | Y: P1.5-1,10-4: F4-1: ST-1655 (cc23) | <65 | 1, 2, 3 |
| 28229 | M13 240267 | UK [England] | 2013 | Y: P1.5-1,10-1: F4-1: ST-1655 (cc23) | <65 | 1 |
| 28231 | M13 240381 | UK [England] | 2013 | Y: P1.5-1,10-4: F4-1: ST-23 (cc23) | >=65 | 2 |
| 28233 | M13 240396 | UK [England] | 2013 | Y: P1.7-2,13-1: F4-1: ST-4446 (cc23) | <65 | 1, 2, 3 |
| 28234 | M13 240407 | UK [England] | 2013 | Y: P1.5-1,10-1: F4-1: ST-1655 (cc23) | <65 | 1, 2 |
| 28235 | M13 240439 | UK [England] | 2013 | Y: P1.5-1,10-1: F4-1: ST-1655 (cc23) | >=65 | 2, 3 |
| 29221 | IBD-524 | Norway | 2011 | Y: P1.5-1,2-2: F5-8: ST-23 (cc23) |  |  |
| 29343 | 32375 | South Africa | 2011 | Y: P1.5-2,10-1: F4-1: ST-4245 (cc23) |  |  |
| 30211 | M13 240735 | UK [England] | 2013 | Y: P1.5-1,10-4: F4-1: ST-23 (cc23) | <65 | 1, 3 |
| 30620 | IBD-480 | Austria | 2012 | Y: P1.5-2,10-1: F4-1: ST-23 (cc23) |  |  |
| 34502 | M13 240484 | UK [England] | 2013 | Y: P1.5-1,10-4: F4-1: ST-1655 (cc23) | >=65 | 1 |
| 34503 | M13 240485 | UK [England] | 2013 | Y: P1.5-1,10-4: F4-1: ST-1655 (cc23) | >=65 | 1, 3 |
| 34618 | NM115 | USA | 1996 | Y: P1.5-1,2-2: F5-8: ST-23 (cc23) |  |  |
| 34627 | NM165 | USA | 1997 | Y: P1.5-2,10-1: F4-1: ST-23 (cc23) |  |  |
| 34632 | NM27 | USA | 1993 | Y: P1.5-1,2-2: F5-8: ST-1622 (cc23) |  |  |
| 34633 | NM271 | USA | 2001 | Y: P1.5-1,2-2: F5-8: ST-23 (cc23) |  |  |
| 34644 | NM3144 | USA | 2009 | Y: P1.5-1,2-2: F-ND: ST-6799 (cc23) |  |  |
| 34645 | NM3158 | USA | 2009 | Y: P1.5-1,2-2: F-ND: ST-6800 (cc23) |  |  |
| 34666 | NM80 | USA | 1995 | Y: P1.5-2,10-1: F4-1: ST-1621 (cc23) |  |  |
| 34668 | NM90 | USA | 1995 | Y: P1.5-1,2-2: F5-8: ST-1622 (cc23) |  |  |
| 35359 | 12.2412.W | UK [Scotland] | 2012 | Y: P1.5-1,2-2: F5-8: ST-23 (cc23) |  |  |
| 35376 | 12.8408.J | UK [Scotland] | 2012 | Y: P1.5-1,10-1: F4-1: ST-1655 (cc23) |  |  |
| 35424 | M13 240529 | UK [England] | 2013 | Y: P1.5-1,10-4: F4-1: ST-1655 (cc23) | <65 | 2 |
| 35432 | M13 240543 | UK [England] | 2013 | Y: P1.5-1,10-1: F4-1: ST-1655 (cc23) | <65 | 1, 3 |
| 35435 | M13 240549 | UK [England] | 2013 | Y: P1.5-1,10-1: F4-1: ST-1655 (cc23) | >=65 | 1, 2, 3 |
| 35445 | M13 240573 | UK [England] | 2013 | Y: P1.5-1,10-1: F4-1: ST-9842 (cc23) | <65 | 3 |
| 35446 | M13 240574 | UK [England] | 2013 | Y: P1.5-1,10-1: F4-1: ST-1655 (cc23) | <65 | 2, 3 |
| 35456 | M13 240599 | UK [Northern Ireland] | 2013 | Y: P1.5-2,10-1: F4-1: ST-23 (cc23) | <65 | 1, 3 |
| 35460 | M13 240610 | UK [England] | 2013 | Y: P1.5-1,10-1: F4-1: ST-1655 (cc23) | <65 | 1, 3 |
| 35468 | M13 240622 | UK [England] | 2013 | Y: P1.5-1,10-1: F4-1: ST-1655 (cc23) | <65 | 1, 2 |
| 35484 | M13 240652 | UK [England] | 2013 | Y: P1.5-1,10-1: F4-1: ST-1655 (cc23) | <65 | 1, 2, 3 |
| 35487 | M13 240663 | UK [England] | 2013 | Y: P1.5-1,10-1: F-ND: ST-1655 (cc23) | <65 | 1, |
| 35491 | M13 240673 | UK [England] | 2013 | Y: P1.5-1,10-4: F1-7: ST-23 (cc23) | <65 | 3 |
| 35508 | M13 240706 | UK [England] | 2013 | Y: P1.5-1,10-4: F4-1: ST-23 (cc23) | >=65 | 1, 2 |
| 35513 | M13 240716 | UK [England] | 2013 | Y: P1.5-1,10-4: F4-1: ST-23 (cc23) | >=65 | 1, 3 |
| 35518 | M13 240721 | UK [England] | 2013 | Y: P1.5-1,10-1: F4-1: ST-1655 (cc23) | >=65 | 2 |
| 35520 | M13 240725 | UK [England] | 2013 | Y: P1.5-1,10-4: F4-1: ST-1655 (cc23) | <65 | 3 |
| 35535 | M13 240749 | UK [England] | 2013 | Y: P1.5-1,10-1: F4-1: ST-1655 (cc23) | >=65 | 1, 2,3 |
| 35536 | M13 240750 | UK [England] | 2013 | Y: P1.5-1,10-1: F4-1: ST-1655 (cc23) | <65 | 3 |
| 35537 | M13 240751 | UK [England] | 2013 | Y: P1.5-1,10-1: F4-1: ST-1655 (cc23) | <65 | 3 |
| 35538 | M13 240752 | UK [England] | 2013 | Y: P1.5-2,10-1: F4-1: ST-23 (cc23) | <65 | 1, 2 |
| 35543 | M14 240010 | UK [England] | 2014 | Y: P1.5-1,10-4: F4-1: ST-23 (cc23) | <65 | 1, |
| 35545 | M14 240012 | UK [England] | 2014 | Y: P1.5-1,10-1: F4-1: ST-1655 (cc23) | >=65 | 1, 2 |
| 35565 | M14 240049 | UK [England] | 2014 | Y: P1.5-1,10-4: F4-1: ST-1655 (cc23) | <65 | 2 |
| 35567 | M14 240056 | UK [England] | 2014 | Y: P1.5-1,10-1: F4-1: ST-1655 (cc23) | >=65 | 1, 2, 3 |
| 35569 | M14 240060 | UK [England] | 2014 | Y: P1.5-1,10-1: F4-1: ST-1655 (cc23) | <65 | 1, 3 |
| 35574 | M14 240075 | UK [England] | 2014 | Y: P1.5-1,10-1: F4-1: ST-1655 (cc23) | >=65 | 2, 3 |
| 35582 | M14 240091 | UK [England] | 2014 | Y: P1.5-1,10-1: F4-1: ST-1655 (cc23) | >=65 | 1 |
| 35590 | M14 240104 | UK [England] | 2014 | Y: P1.5-1,10-4: F4-1: ST-23 (cc23) | >=65 | 1, 2 |
| 35591 | M14 240105 | UK [England] | 2014 | Y: P1.5-2,10-1: F4-1: ST-23 (cc23) | <65 | 1, 2, 3 |
| 35592 | M14 240108 | UK [Wales] | 2014 | Y: P1.5-1,10-1: F4-1: ST-1655 (cc23) | <65 | 3 |
| 35595 | M14 240111 | UK [England] | 2014 | Y: P1.5-1,10-1: F4-1: ST-1655 (cc23) | >=65 | 3 |
| 35596 | M14 240112 | UK [England] | 2014 | Y: P1.5-1,10-4: F4-1: ST-1655 (cc23) | >=65 | 3 |
| 35599 | M14 240117 | UK [England] | 2014 | Y: P1.5-1,10-4: F4-1: ST-1655 (cc23) | >=65 | 2, 3 |
| 35600 | M14 240118 | UK [England] | 2014 | Y: P1.5-2,10-1: F4-1: ST-23 (cc23) | <65 | 3 |
| 35615 | M14 240136 | UK [England] | 2014 | Y: P1.5-1,10-10: F4-1: ST-1655 (cc23) | <65 | 1 |
| 35616 | M14 240140 | UK [England] | 2014 | Y: P1.5-1,10-4: F4-1: ST-23 (cc23) | <65 | 1, 3 |
| 35620 | M14 240144 | UK [England] | 2014 | Y: P1.5-1,10-1: F4-1: ST-1655 (cc23) | <65 | 3 |
| 35627 | M14 240157 | UK [England] | 2014 | Y: P1.5-1,10-1: F4-1: ST-1655 (cc23) | >=65 | 3 |
| 35637 | M14 240202 | UK [England] | 2014 | Y: P1.5-1,10-10: F4-1: ST-11280 (cc23) | >=65 | 1, 2, 3 |
| 35642 | M14 240210 | UK [England] | 2014 | Y: P1.5-1,10-4: F4-1: ST-23 (cc23) | >=65 | 1, 2 |
| 35653 | M14 240225 | UK [England] | 2014 | Y: P1.5-1,10-10: F4-1: ST-1655 (cc23) | >=65 | 1, 3 |
| 35660 | M14 240237 | UK [Wales] | 2014 | Y: P1.5-1,10-1: F4-1: ST-1655 (cc23) | >=65 | 1 |
| 35665 | M14 240246 | UK [England] | 2014 | Y: P1.5-2,10-1: F4-1: ST-11293 (cc23) | >=65 | 3 |
| 35684 | M14 240276 | UK [England] | 2014 | Y: P1.5-1,10-1: F4-1: ST-1655 (cc23) | >=65 | 2, 3 |
| 35692 | M14 240285 | UK [England] | 2014 | Y: P1.5-1,10-1: F4-1: ST-1655 (cc23) | <65 | 2 |
| 35694 | M14 240287 | UK [England] | 2014 | Y: P1.5-1,10-1: F4-1: ST-1655 (cc23) | <65 | 1, 3 |
| 35696 | M14 240291 | UK [England] | 2014 | Y: P1.5-1,10-4: F4-1: ST-1655 (cc23) | <65 | 1, 2 |
| 35709 | M14 240313 | UK [England] | 2014 | W/Y: P1.5-1,10-1: F4-1: ST-1655 (cc23) | >=65 | 2, 3 |
| 35721 | M14 240335 | UK [England] | 2014 | Y: P1.5-1,10-1: F4-1: ST-11331 (cc23) | <65 | 1, 2 |
| 35746 | M14 240378 | UK [Wales] | 2014 | Y: P1.5-1,10-4: F4-1: ST-1655 (cc23) | >=65 | 1 |
| 35761 | M14 240404 | UK [England] | 2014 | Y: P1.5-2,10-1: F5-8: ST-23 (cc23) | <65 | 2 |
| 35767 | M14 240423 | UK [England] | 2014 | Y: P1.5-1,10-1: F4-1: ST-1655 (cc23) | <65 | 1, 2, 3 |
| 35802 | M14 240487 | UK [England] | 2014 | Y: P1.5-1,10-4: F4-1: ST-1655 (cc23) | <65 | 2 |
| 35804 | M14 240489 | UK [England] | 2014 | Y: P1.5-1,10-1: F4-1: ST-1655 (cc23) | >=65 | 2 |
| 35830 | M13 240674 | UK [England] | 2013 | Y: P1.5-1,10-1: F4-1: ST-1655 (cc23) | <65 | 1, 2, 3 |
| 36316 | 1459000337 | Sweden | 2014 | Y: P1.5-2,10-1: F4-1: ST-11293 (cc23) |  |  |
| 36829 | IE15Nm15 | Ireland | 2015 | Y: P1.5-1,10-1: F4-1: ST-1655 (cc23) |  |  |
| 37664 | M14 240156 | UK [England] | 2014 | Y: P1.5-1,10-1: F4-1: ST-1655 (cc23) | <65 | 2, 3 |
| 37666 | M14 240170 | UK [England] | 2014 | Y: P1.5-2,10-1: F5-12: ST-23 (cc23) | >=65 | 3 |
| 37667 | M14 240172 | UK [England] | 2014 | Y: P1.5-1,10-1: F4-1: ST-1655 (cc23) | >=65 | 1, 3 |
| 37672 | M14 240501 | UK [England] | 2014 | Y: P1.5-1,10-1: F4-1: ST-1655 (cc23) | >=65 | 1, 2, 3 |
| 37689 | M14 240521 | UK [England] | 2014 | Y: P1.5-1,10-1: F4-1: ST-11866 (cc23) | >=65 | 2, 3 |
| 37691 | M14 240523 | UK [England] | 2014 | Y: P1.5-1,2-2: F5-8: ST-23 (cc23) | <65 | 3 |
| 37694 | M14 240526 | UK [England] | 2014 | Y: P1.5-1,10-1: F4-1: ST-1655 (cc23) | >=65 | 3 |
| 37697 | M14 240530 | UK [England] | 2014 | Y: P1.5-1,2-2: F5-8: ST-3582 (cc23) | <65 | 1, 3 |
| 37703 | M14 240536 | UK [England] | 2014 | Y: P1.5-1,10-1: F4-1: ST-1655 (cc23) | <65 | 2, 3 |
| 37714 | M14 240553 | UK [England] | 2014 | Y: P1.5-1,10-1: F4-1: ST-1655 (cc23) | >=65 | 1, 3 |
| 37751 | M14 240607 | UK [England] | 2014 | Y: P1.5-2,10-1: F5-12: ST-23 (cc23) | >=65 | 2, 3 |
| 37765 | M14 240629 | UK [England] | 2014 | Y: P1.5-1,2-2: F5-8: ST-11868 (cc23) | >=65 | 2, 3 |
| 37768 | M14 240632 | UK [England] | 2014 | Y: P1.5-1,10-4: F4-1: ST-11472 (cc23) | >=65 | 1, 2 |
| 37771 | M14 240635 | UK [England] | 2014 | Y: P1.5-1,10-1: F4-1: ST-1655 (cc23) | <65 | 1, 3 |
| 37794 | M15 240006 | UK [England] | 2015 | Y: P1.5-1,10-1: F4-1: ST-1655 (cc23) | >=65 | 1, 3 |
| 37795 | M15 240007 | UK [Wales] | 2015 | Y: P1.5-1,2-2: F5-8: ST-23 (cc23) | <65 | 2, 3 |
| 37796 | M15 240009 | UK [England] | 2015 | Y: P1.5-1,10-4: F4-1: ST-23 (cc23) | >=65 | 1, 3 |
| 37812 | M15 240030 | UK [England] | 2015 | Y: P1.5-1,10-1: F4-1: ST-1655 (cc23) | >=65 | 2, 3 |
| 37820 | M15 240040 | UK [England] | 2015 | Y: P1.5-1,10-1: F4-1: ST-1655 (cc23) | <65 | 1 |
| 37827 | M15 240047 | UK [England] | 2015 | Y: P1.5-1,10-4: F4-1: ST-23 (cc23) | <65 | 3 |
| 37833 | M15 240054 | UK [England] | 2015 | Y: P1.5-1,10-1: F4-1: ST-1655 (cc23) | >=65 | 1 |
| 37836 | M15 240057 | UK [England] | 2015 | Y: P1.5-1,10-1: F4-1: ST-1655 (cc23) | <65 | 2 |
| 37838 | M15 240059 | UK [England] | 2015 | Y: P1.5-1,10-4: F4-1: ST-1655 (cc23) | <65 | 2 |
| 37841 | M15 240062 | UK [England] | 2015 | Y: P1.5-2,10-1: F4-1: ST-23 (cc23) | <65 | 3 |
| 37843 | M15 240064 | UK [England] | 2015 | Y: P1.5-1,10-4: F4-1: ST-1655 (cc23) | <65 | 1 |
| 37847 | M15 240068 | UK [England] | 2015 | Y: P1.5-2,10-1: F4-1: ST-23 (cc23) | <65 | 3 |
| 37850 | M15 240072 | UK [England] | 2015 | Y: P1.5-1,2-2: F5-8: ST-23 (cc23) | <65 | 2 |
| 37853 | M15 240077 | UK [England] | 2015 | Y: P1.5-2,10-1: F4-1: ST-23 (cc23) | <65 | 1 |
| 37858 | M15 240082 | UK [England] | 2015 | Y: P1.5-1,10-1: F4-1: ST-1655 (cc23) | >=65 | 1, 2, 3 |
| 37859 | M15 240083 | UK [England] | 2015 | Y: P1.5-1,10-1: F4-1: ST-1655 (cc23) | >=65 | 1, 3 |
| 37867 | M15 240098 | UK [England] | 2015 | Y: P1.5-1,10-4: F4-1: ST-23 (cc23) | <65 | 1, 2 |
| 37869 | M15 240100 | UK [England] | 2015 | Y: P1.5-1,10-1: F4-1: ST-1655 (cc23) | <65 | 3 |
| 37885 | M15 240119 | UK [England] | 2015 | Y: P1.5-1,10-1: F4-1: ST-1655 (cc23) | <65 | 3 |
| 37889 | M15 240126 | UK [England] | 2015 | Y: P1.5-1,10-1: F4-1: ST-1655 (cc23) | <65 | 1, 3 |
| 37892 | M15 240129 | UK [England] | 2015 | NG: P1.5-1,2-2: F5-8: ST-23 (cc23) | >=65 | 1, 3 |
| 37895 | M15 240132 | UK [England] | 2015 | Y: P1.5-1,10-1: F4-1: ST-1655 (cc23) | <65 | 1, 2, 3 |
| 37897 | M15 240134 | UK [England] | 2015 | Y: P1.5-1,10-1: F4-1: ST-1655 (cc23) | <65 | 2 |
| 37899 | M15 240136 | UK [England] | 2015 | Y: P1.5-1,10-1: F4-1: ST-1655 (cc23) | <65 | 1 |
| 37911 | M15 240152 | UK [England] | 2015 | Y: P1.5-1,10-1: F4-1: ST-1655 (cc23) | <65 | 1, 3 |
| 37914 | M15 240157 | UK [England] | 2015 | W: P1.5-1,10-1: F4-1: ST-1655 (cc23) | >=65 | 1, 2 |
| 37917 | M15 240160 | UK [England] | 2015 | Y: P1.5-1,10-10: F4-1: ST-1655 (cc23) | <65 | 2, 3 |
| 37923 | M15 240174 | UK [England] | 2015 | Y: P1.5-1,10-1: F4-1: ST-1655 (cc23) | <65 | 1, 2 |
| 37926 | M15 240177 | UK [England] | 2015 | Y: P1.5-1,10-1: F4-1: ST-10458 (cc23) | >=65 | 2, 3 |
| 37938 | M15 240191 | UK [England] | 2015 | Y: P1.5-2,10-1: F4-1: ST-23 (cc23) | >=65 | 1 |
| 37939 | M15 240192 | UK [England] | 2015 | Y: P1.5-1,10-1: F4-1: ST-1655 (cc23) | <65 | 1, 2 |
| 37941 | M15 240194 | UK [England] | 2015 | Y: P1.5-1,10-1: F4-1: ST-1655 (cc23) | >=65 | 1, 2, 3 |
| 37944 | M15 240199 | UK [England] | 2015 | Y: P1.5-1,10-1: F4-1: ST-1655 (cc23) | <65 | 1, 2 |
| 37955 | M15 240212 | UK [England] | 2015 | Y: P1.5-1,10-1: F4-1: ST-1655 (cc23) | >=65 | 1, 2 |
| 37963 | M15 240223 | UK [England] | 2015 | Y: P1.5-2,10-1: F5-12: ST-23 (cc23) | <65 | 1, 3 |
| 37979 | M15 240246 | UK [England] | 2015 | Y: P1.5-1,2-2: F5-8: ST-4183 (cc23) | >=65 | 1 |
| 37980 | M15 240247 | UK [England] | 2015 | Y: P1.5-1,10-1: F4-1: ST-1655 (cc23) | <65 | 1, 3 |
| 37982 | M15 240250 | UK [England] | 2015 | Y: P1.5-1,10-1: F4-1: ST-1655 (cc23) | >=65 | 2, 3 |
| 37993 | M15 240265 | UK [England] | 2015 | Y: P1.5-1,10-1: F4-1: ST-1655 (cc23) | >=65 | 1, 2, 3 |
| 38005 | M15 240285 | UK [England] | 2015 | Y: P1.5-1,10-1: F4-1: ST-1655 (cc23) | >=65 | 3 |
| 38010 | M15 240290 | UK [England] | 2015 | Y: P1.5-1,10-1: F4-1: ST-1655 (cc23) | <65 | 1 |
| 38012 | M15 240292 | UK [England] | 2015 | Y: P1.5-1,10-4: F4-1: ST-23 (cc23) | <65 | 1, 2, 3 |
| 38013 | M15 240294 | UK [England] | 2015 | Y: P1.5-1,10-4: F4-1: ST-1655 (cc23) | >=65 | 1, 2 |
| 38014 | M15 240295 | UK [England] | 2015 | Y: P1.5-1,10-1: F4-1: ST-1655 (cc23) | <65 | 1 |
| 38021 | M15 240305 | UK [England] | 2015 | Y: P1.5-1,10-1: F4-1: ST-1655 (cc23) | <65 | 1, 2, 3 |
| 38028 | M15 240319 | UK [England] | 2015 | Y: P1.5-1,10-1: F4-1: ST-1655 (cc23) | >=65 | 1, 3 |
| 38053 | M15 240352 | UK [England] | 2015 | Y: P1.5-1,10-4: F4-1: ST-11875 (cc23) | <65 | 1, 2 |
| 38078 | M15 240440 | UK [England] | 2015 | Y: P1.5-1,10-1: F4-1: ST-1655 (cc23) | <65 | 1 |
| 38087 | M15 240464 | UK [England] | 2015 | Y: P1.5-1,2-2: F5-8: ST-11490 (cc23) | <65 | 3 |
| 38099 | M15 240498 | UK [England] | 2015 | Y: P1.5-1,10-1: F4-1: ST-1655 (cc23) | <65 | 1, 3 |
| 38104 | M15 240525 | UK [England] | 2015 | Y: P1.5-1,10-1: F4-1: ST-1655 (cc23) | <65 | 2 |
| 38116 | M15 240600 | UK [England] | 2015 | Y: P1.5-1,10-1: F4-1: ST-1655 (cc23) | <65 | 1, 2 |
| 38118 | M14 240523b | UK [England] | 2014 | Y: P1.5-1,2-2: F5-8: ST-23 (cc23) |  |  |
| 38157 | 93Mo | Slovenia | 2014 | Y: P1.5-1,2-5: F5-8: ST-23 (cc23) |  |  |
| 38908 | P55 | Italy | 2012 | Y: P1.5-2,10-25: F-ND: ST-23 (cc23) |  |  |
| 38934 | P97 | Italy | 2013 | A: P1.5-2,10-2: F1-21: ST-11131 (cc23) |  |  |
| 38935 | P98 | Italy | 2013 | Y: P1.5-2,10-2: F1-55: ST-23 (cc23) |  |  |
| 38952 | P116 | Italy | 2013 | Y: P1.5-2,10-36: F5-88: ST-23 (cc23) |  |  |
| 39321 | M15 240631 | UK [England] | 2015 | W/Y: P1.5-1,10-1: F4-1: ST-1655 (cc23) | <65 | 3 |
| 39330 | M15 240642 | UK [England] | 2015 | Y: P1.5-2,10-1: F4-1: ST-23 (cc23) | <65 | 2, 3 |
| 39332 | M15 240644 | UK [Wales] | 2015 | Y: P1.5-1,10-1: F4-1: ST-1655 (cc23) | >=65 | 2, 3 |
| 39339 | M15 240653 | UK [England] | 2015 | Y: P1.5-2,10-2: F2-13: ST-9253 (cc23) | >=65 | 1, 2, 3 |
| 39347 | M15 240697 | UK [England] | 2015 | Y: P1.5-1,10-1: F4-1: ST-11754 (cc23) | >=65 | 1, 3 |
| 39359 | M15 240723 | UK [England] | 2015 | Y: P1.5-1,10-4: F4-1: ST-23 (cc23) | <65 | 1, 2, 3 |
| 39363 | M15 240728 | UK [England] | 2015 | Y: P1.5-1,10-1: F4-1: ST-1655 (cc23) | >=65 | 1, 3 |
| 39376 | M15 240749 | UK [England] | 2015 | Y: P1.5-1,10-1: F4-1: ST-1655 (cc23) | <65 | 1, 2 |
| 39379 | M15 240752 | UK [England] | 2015 | Y: P1.5-1,10-1: F4-1: ST-1655 (cc23) | >=65 | 1, 2, 3 |
| 39419 | M15 240807 | UK [England] | 2015 | Y: P1.5-1,10-1: F4-1: ST-1655 (cc23) | <65 | 3 |
| 39420 | M15 240808 | UK [England] | 2015 | Y: P1.5-1,10-1: F4-1: ST-1655 (cc23) | >=65 | 1 |
| 39426 | M15 240822 | UK [Wales] | 2015 | Y: P1.5-1,10-1: F4-1: ST-11754 (cc23) | <65 | 1, 2, 3 |
| 39432 | M15 240832 | UK [England] | 2015 | Y: P1.5-1,10-1: F4-1: ST-1655 (cc23) | <65 | 1, 2, 3 |
| 39437 | M15 240847 | UK [England] | 2015 | Y: P1.5-1,10-1: F4-1: ST-1655 (cc23) | >=65 | 1 |
| 39440 | M15 240850 | UK [England] | 2015 | Y: P1.5-1,10-1: F4-1: ST-1655 (cc23) | <65 | 2, 3 |
| 40206 | LNP28265 | France | 2015 | Y: P1.5-1,2-2: F5-8: ST-3582 (cc23) |  |  |
| 40209 | LNP28268 | France | 2015 | Y: P1.5-2,10-1: F5-12: ST-23 (cc23) |  |  |
| 40289 | 12-588 | Sweden |  | Y: P1.5-1,2-2: F5-8: ST-23 (cc23) |  |  |
| 40290 | LNP28352 | France | 2015 | Y: P1.5-1,10-1: F4-1: ST-1655 (cc23) |  |  |
| 40292 | 05-231 | Sweden |  | Y: P1.5-2,10-1: F1-7: ST-2692 (cc23) |  |  |
| 40326 | 2606 | Italy | 2014 | Y: P1.5-2,10-2: F4-1: ST-23 (cc23) |  |  |
| 40328 | 2631 | Italy | 2015 | Y: P1.5-2,10-2: F1-68: ST-23 (cc23) |  |  |
| 40335 | 2558 | Italy | 2014 | Y: P1.5-2,10-2: F2-13: ST-23 (cc23) |  |  |
| 40346 | 2642 | Italy | 2015 | Y: P1.5-2,10-2: F4-1: ST-23 (cc23) |  |  |
| 40351 | 2681 | Italy | 2015 | Y: P1.5-2,10-2: F2-13: ST-23 (cc23) |  |  |
| 40356 | 2700 | Italy | 2015 | Y: P1.5-2,10-2: F2-13: ST-23 (cc23) |  |  |
| 40359 | 2713 | Italy | 2015 | Y: P1.5-2,10-2: F2-13: ST-23 (cc23) |  |  |
| 40361 | 2726 | Italy | 2015 | Y: P1.5-2,10-2: F2-13: ST-23 (cc23) |  |  |
| 40362 | 2732 | Italy | 2015 | Y: P1.5-2,10-2: F5-17: ST-23 (cc23) |  |  |
| 40405 | LNP28380 | France | 2016 | Y: P1.5-1,2-2: F5-8: ST-23 (cc23) |  |  |
| 41312 | 1359000130 | Sweden | 2013 | Y: P1.5-2,10-1: F4-1: ST-23 (cc23) |  |  |
| 41330 | 1459000362 | Sweden | 2014 | Y: P1.5-2,10-1: F4-1: ST-23 (cc23) |  |  |
| 41466 | M15 240870 | UK [England] | 2015 | Y: P1.5-1,10-1: F4-1: ST-1655 (cc23) | <65 | 1, 2 |
| 41476 | M15 240882 | UK [England] | 2015 | Y: P1.5-2,10-1: F4-1: ST-23 (cc23) | <65 | 2 |
| 41480 | M15 240887 | UK [England] | 2015 | Y: P1.5-1,10-1: F4-1: ST-1655 (cc23) | <65 | 1 |
| 41495 | M15 240911 | UK [England] | 2015 | Y: P1.5-1,10-1: F4-1: ST-1655 (cc23) | <65 | 1, 2, 3 |
| 41497 | M15 240913 | UK [England] | 2015 | Y: P1.5-2,10-1: F4-1: ST-23 (cc23) | <65 | 3 |
| 41498 | M15 240914 | UK [Wales] | 2015 | Y: P1.5-1,10-1: F4-1: ST-1655 (cc23) | <65 | 1, 3 |
| 41499 | M15 240915 | UK [England] | 2015 | Y: P1.5-1,10-1: F4-1: ST-1655 (cc23) | >=65 | 1, 2 |
| 41508 | M15 240926 | UK [England] | 2015 | Y: P1.5-1,10-1: F4-1: ST-1655 (cc23) | >=65 | 1 |
| 41514 | M15 240935 | UK [England] | 2015 | Y: P1.5-1,10-4: F4-1: ST-1655 (cc23) | >=65 | 1, 3 |
| 41517 | M15 240938 | UK [Wales] | 2015 | Y: P1.5-1,10-1: F4-1: ST-1655 (cc23) | >=65 | 1, 3 |
| 41521 | M15 240945 | UK [England] | 2015 | Y: P1.5-1,10-1: F4-1: ST-1655 (cc23) | >=65 | 2 |
| 41529 | M15 240957 | UK [England] | 2015 | Y: P1.18-1,30-2: F4-1: ST-23 (cc23) | >=65 | 2, 3 |
| 41534 | M15 240965 | UK [England] | 2015 | Y: P1.5-1,10-1: F4-1: ST-1655 (cc23) | >=65 | 1, 3 |
| 41535 | M15 240967 | UK [England] | 2015 | Y: P1.5-2,10-1: F4-1: ST-23 (cc23) | <65 | 1 |
| 41553 | M15 240989 | UK [England] | 2015 | Y: P1.5-1,10-1: F4-1: ST-1655 (cc23) | >=65 | 2 |
| 41556 | M15 240992 | UK [England] | 2015 | Y: P1.5-1,10-4: F4-1: ST-23 (cc23) | <65 | 3 |
| 41557 | M15 240994 | UK [England] | 2015 | Y: P1.5-1,10-1: F4-1: ST-1655 (cc23) | <65 | 2, 3 |
| 41674 | LNP28430 | France | 2016 | Y: P1.5-2,10-1: F4-1: ST-23 (cc23) |  |  |
| 41736 | LNP27751 | France | 2014 | Y: P1.5-2,10-1: F4-1: ST-23 (cc23) |  |  |
| 41742 | LNP27841 | France | 2014 | Y: P1.5-1,10-1: F4-1: ST-1655 (cc23) |  |  |
| 41754 | LNP27987 | France | 2015 | Y: P1.5-2,10-2: F4-1: ST-3030 (cc23) |  |  |
| 41769 | LNP28081 | France | 2015 | Y: P1.5-2,ND: F2-13: ST-23 (cc23) |  |  |
| 41773 | LNP28096 | France | 2015 | Y: P1.5-2,10-2: F-ND: ST-23 (cc23) |  |  |
| 41806 | LNP28453 | France | 2016 | Y: P1.5-1,2-2: F5-8: ST-23 (cc23) |  |  |
| 41967 | 16-101 | Sweden | 2016 | Y: P1.5-2,10-1: F4-1: ST-23 (cc23) |  |  |
| 42048 | LNP28482 | France | 2016 | Y: P1.5-2,10-1: F5-12: ST-23 (cc23) |  |  |
| 42370 | 1459000219 | Sweden | 2014 | Y: P1.5-1,2-2: F5-8: ST-23 (cc23) |  |  |
| 42426 | 15-405 | Sweden | 2015 | Y: P1.5-2,10-1: F4-1: ST-23 (cc23) |  |  |
| 42434 | 16-56 | Sweden | 2016 | Y: P1.5-1,2-2: F5-8: ST-23 (cc23) |  |  |
| 42474 | M16 240002 | UK [England] | 2016 | Y: P1.5-1,10-4: F4-1: ST-23 (cc23) | <65 | 1 |
| 42479 | M16 240009 | UK [England] | 2016 | Y: P1.5-1,10-1: F4-1: ST-1655 (cc23) | <65 | 3 |
| 42487 | M16 240018 | UK [England] | 2016 | Y: P1.5-1,10-1: F4-1: ST-1655 (cc23) | >=65 | 3 |
| 42490 | M16 240021 | UK [England] | 2016 | Y: P1.5-1,10-1: F4-1: ST-1655 (cc23) | <65 | 2 |
| 42493 | M16 240026 | UK [England] | 2016 | W: P1.5-1,10-1: F4-1: ST-1655 (cc23) | >=65 | 1, 2 |
| 42496 | M16 240030 | UK [England] | 2016 | Y: P1.5-1,10-1: F4-1: ST-1655 (cc23) | >=65 | 1, 3 |
| 42518 | M16 240053 | UK [England] | 2016 | Y: P1.5-1,10-1: F4-1: ST-12176 (cc23) | <65 | 3 |
| 42527 | M16 240067 | UK [Wales] | 2016 | Y: P1.5-1,10-1: F5-91: ST-1655 (cc23) | >=65 | 1, 2 |
| 42529 | M16 240069 | UK [England] | 2016 | Y: P1.5-1,10-1: F4-1: ST-1655 (cc23) | <65 | 2, 3 |
| 42538 | M16 240081 | UK [Northern Ireland] | 2016 | Y: P1.7-2,30: F4-1: ST-1655 (cc23) | >=65 | 1, 3 |
| 42539 | M16 240082 | UK [England] | 2016 | Y: P1.5-1,2-2: F5-8: ST-3582 (cc23) | >=65 | 1, 2 |
| 42557 | M16 240118 | UK [England] | 2016 | Y: P1.5-2,10-1: F5-8: ST-23 (cc23) | >=65 | 2, 3 |
| 42574 | M16 240139 | UK [England] | 2016 | Y: P1.5-1,10-1: F4-1: ST-1655 (cc23) | <65 | 1, 3 |
| 42582 | M16 240148 | UK [England] | 2016 | Y: P1.5-1,10-1: F4-1: ST-1655 (cc23) | >=65 | 2, 3 |
| 42593 | M16 240161 | UK [England] | 2016 | Y: P1.5-2,10-1: F4-1: ST-23 (cc23) | <65 | 1, 3 |
| 42599 | M16 240170 | UK [Wales] | 2016 | Y: P1.5-1,2-2: F5-8: ST-23 (cc23) | <65 | 1, 2, 3 |
| 42600 | M16 240173 | UK [England] | 2016 | Y: P1.5-1,10-4: F4-1: ST-1655 (cc23) | <65 | 2 |
| 42607 | M16 240182 | UK [England] | 2016 | Y: P1.5-1,10-4: F4-1: ST-1655 (cc23) | <65 | 2, 3 |
| 42608 | M16 240184 | UK [England] | 2016 | Y: P1.5-1,10-1: F4-1: ST-1655 (cc23) | <65 | 1, 2 |
| 42609 | M16 240185 | UK [England] | 2016 | Y: P1.5-1,10-1: F4-1: ST-1655 (cc23) | <65 | 1 |
| 42613 | M16 240190 | UK [England] | 2016 | Y: P1.5-2,10-1: F4-1: ST-23 (cc23) | >=65 | 1, 3 |
| 42760 | LNP28536 | France | 2016 | Y: P1.5-1,2-59: F5-8: ST-23 (cc23) |  |  |
| 44106 | ST41616 | UK [England] | 2015 | Y: P1.5-1,2-2: F5-8: ST-3582 (cc23) |  |  |
| 44189 | ST41154 | UK [England] | 2015 | Y: P1.5-1,10-10: F4-1: ST-12353 (cc23) |  |  |
| 44216 | GL41142 | UK [Scotland] | 2014 | Y: P1.5-1,10-1: F4-1: ST-1655 (cc23) |  |  |
| 44365 | ST40485 | UK [England] | 2015 | Y: P1.5-2,10-2: F4-1: ST-183 (cc23) |  |  |
| 44439 | OX42156 | UK [England] | 2015 | Y: P1.5-2,10-2: F2-13: ST-23 (cc23) |  |  |
| 44519 | OX40944 | UK [England] | 2015 | Y: P1.5-2,10-2: F4-1: ST-10833 (cc23) |  |  |
| 44551 | OX41306 | UK [England] | 2015 | Y: P1.5-1,2-2: F5-8: ST-23 (cc23) |  |  |
| 44561 | OX41460 | UK [England] | 2015 | Y: P1.5-1,10-4: F4-1: ST-23 (cc23) |  |  |
| 44577 | OX41591 | UK [England] | 2015 | Y: P1.5-2,10-2: F1-68: ST-23 (cc23) |  |  |
| 44626 | OX42130 | UK [England] | 2015 | Y: P1.5-2,10-1: F4-1: ST-23 (cc23) |  |  |
| 44627 | OX42147 | UK [England] | 2015 | Y: P1.5-2,10-2: F1-68: ST-23 (cc23) |  |  |
| 44639 | M16 240233 | UK [England] | 2016 | Y: P1.5-1,10-4: F4-1: ST-1655 (cc23) | >=65 | 3 |
| 44706 | M16 240217 | UK [England] | 2016 | Y: P1.5-1,10-1: F4-1: ST-11754 (cc23) | >=65 | 1 |
| 44716 | M16 240236 | UK [England] | 2016 | Y: P1.5-1,10-1: F4-1: ST-1655 (cc23) | >=65 | 3 |
| 44720 | M16 240241 | UK [England] | 2016 | Y: P1.5-1,10-1: F4-1: ST-1655 (cc23) | >=65 | 3 |
| 44738 | M16 240264 | UK [England] | 2016 | Y: P1.5-1,10-4: F4-1: ST-23 (cc23) | >=65 | 2, 3 |
| 44747 | M16 240278 | UK [England] | 2016 | Y: P1.5-1,10-4: F4-1: ST-23 (cc23) | <65 | 1, 2, 3 |
| 44748 | M16 240280 | UK [England] | 2016 | Y: P1.5-1,10-1: F4-1: ST-1655 (cc23) | >=65 | 1, 2, 3 |
| 44751 | M16 240286 | UK [England] | 2016 | Y: P1.5-1,10-4: F4-1: ST-23 (cc23) | <65 | 1 |
| 44760 | M16 240362 | UK [England] | 2016 | Y: P1.5-2,10-1: F4-1: ST-23 (cc23) | <65 | 1 |
| 44775 | M16 240389 | UK [England] | 2016 | Y: P1.5-1,10-1: F4-1: ST-1655 (cc23) | <65 | 1, 2, 3 |
| 44793 | M16 240413 | UK [England] | 2016 | Y: P1.5-1,10-1: F4-1: ST-1655 (cc23) | >=65 | 1, 2 |
| 44814 | M16 240439 | UK [England] | 2016 | Y: P1.5-1,10-4: F4-1: ST-23 (cc23) | >=65 | 1, 2, 3 |
| 44817 | M16 240445 | UK [England] | 2016 | Y: P1.5-1,2-2: F5-8: ST-1625 (cc23) | >=65 |  |
| 44822 | M16 240492 | UK [England] | 2016 | Y: P1.5-1,10-1: F4-1: ST-1655 (cc23) | >=65 |  |
| 45024 | 16-304 | Sweden | 2016 | Y: P1.5-1,2-2: F5-8: ST-23 (cc23) |  |  |
| 46375 | 16-325 | Sweden | 2016 | Y: P1.5-1,10-1: F4-1: ST-1655 (cc23) |  |  |
| 46545 | GL40066 | UK [Scotland] | 2014 | Y: P1.5-1,10-1: F4-1: ST-1655 (cc23) |  |  |
| 46593 | GL40915 | UK [Scotland] | 2014 | Y: P1.5-1,2-2: F5-8: ST-23 (cc23) |  |  |
| 46595 | GL40926 | UK [Scotland] | 2014 | Y: P1.5-1,2-2: F5-8: ST-23 (cc23) |  |  |
| 46624 | GL41410 | UK [Scotland] | 2014 | Y: P1.5-2,10-2: F4-1: ST-183 (cc23) |  |  |
| 46690 | GL42583 | UK [Scotland] | 2015 | Y: P1.5-1,2-2: F5-8: ST-23 (cc23) |  |  |
| 47169 | 16-371 | Sweden | 2016 | Y: P1.5-2,10-1: F4-1: ST-23 (cc23) |  |  |
| 47171 | 16-390 | Sweden | 2016 | Y: P1.5-1,2-2: F5-8: ST-23 (cc23) |  |  |
| 47259 | M16 240213 | UK [England] | 2016 | Y: P1.5-1,10-1: F4-1: ST-1655 (cc23) | <65 | 1, 3 |
| 47260 | M16 240240 | UK [England] | 2016 | Y: P1.5-1,10-1: F4-1: ST-1655 (cc23) | >=65 | 1, 2, 3 |
| 47270 | M16 240522 | UK [England] | 2016 | Y: P1.5-1,10-1: F4-1: ST-1655 (cc23) | >=65 | 1, 2 |
| 47281 | M16 240536 | UK [England] | 2016 | Y: P1.5-1,10-100: F4-1: ST-1655 (cc23) | >=65 | 2, 3 |
| 47282 | M16 240537 | UK [England] | 2016 | Y: P1.5-1,10-1: F4-1: ST-1655 (cc23) | >=65 | 1, 3 |
| 47283 | M16 240538 | UK [England] | 2016 | Y: P1.5-1,10-1: F4-1: ST-1655 (cc23) | >=65 | 1, 3 |
| 47284 | M16 240539 | UK [England] | 2016 | Y: P1.5-1,10-1: F4-1: ST-1655 (cc23) | >=65 | 2 |
| 47295 | M16 240554 | UK [England] | 2016 | Y: P1.5-1,10-1: F4-1: ST-1655 (cc23) | <65 | 1, 2 |
| 47297 | M16 240563 | UK [England] | 2016 | Y: P1.5-1,10-4: F4-1: ST-1655 (cc23) | >=65 | 2, 3 |
| 47302 | M16 240569 | UK [England] | 2016 | Y: P1.5-1,10-1: F4-1: ST-1655 (cc23) | >=65 | 2, 3 |
| 47315 | M16 240641 | UK [England] | 2016 | Y: P1.5-1,10-1: F4-1: ST-1655 (cc23) | <65 | 1, 3 |
| 47316 | M16 240642 | UK [England] | 2016 | Y: P1.5-1,10-4: F4-1: ST-23 (cc23) | >=65 | 1, 3 |
| 47317 | M16 240643 | UK [England] | 2016 | Y: P1.5-1,10-1: F4-1: ST-1655 (cc23) | >=65 | 2, 3 |
| 47320 | M16 240648 | UK [England] | 2016 | Y: P1.5-1,10-4: F4-1: ST-23 (cc23) | >=65 | 1, 2 |
| 47324 | M16 240655 | UK [England] | 2016 | Y: P1.5-1,10-4: F4-1: ST-23 (cc23) | <65 | 1, 3 |
| 47327 | M16 240659 | UK [England] | 2016 | Y: P1.5-1,10-12: F4-1: ST-1655 (cc23) | <65 | 2, 3 |
| 47330 | M16 240668 | UK [England] | 2016 | Y: P1.5-1,10-1: F4-1: ST-1655 (cc23) | >=65 | 1, 2 |
| 47335 | M16 240673 | UK [England] | 2016 | Y: P1.5-1,10-4: F4-1: ST-1655 (cc23) | >=65 | 3 |
| 49377 | CA40030 | UK [Wales] | 2015 | Y: P1.5-1,10-1: F4-1: ST-1655 (cc23) |  |  |
| 49407 | CA40208 | UK [Wales] | 2015 | Y: P1.5-1,2-2: F5-8: ST-3582 (cc23) |  |  |
| 49447 | CA40769 | UK [Wales] | 2015 | Y: P1.5-1,2-2: F5-8: ST-23 (cc23) |  |  |
| 49448 | CA40795 | UK [Wales] | 2015 | Y: P1.5-1,2-2: F5-8: ST-23 (cc23) |  |  |
| 49462 | CA40875 | UK [Wales] | 2015 | Y: P1.5-2,10-2: F4-1: ST-183 (cc23) |  |  |
| 49654 | CA44695 | UK [Wales] | 2015 | Y: P1.5-1,2-2: F5-8: ST-23 (cc23) |  |  |
| 49673 | GL40874 | UK [Scotland] | 2014 | Y: P1.5-1,10-1: F4-1: ST-1655 (cc23) |  |  |
| 49686 | LO40121 | UK [England] | 2014 | Y: P1.5-1,2-2: F5-8: ST-23 (cc23) |  |  |
| 49687 | LO40132 | UK [England] | 2014 | Y: P1.5-1,2-2: F5-8: ST-23 (cc23) |  |  |
| 49699 | LO40810 | UK [England] | 2015 | Y: P1.5-1,2-2: F5-8: ST-23 (cc23) |  |  |
| 49701 | LO40814 | UK [England] | 2015 | Y: P1.5-1,2-2: F5-8: ST-23 (cc23) |  |  |
| 49730 | CM40369 | UK [England] | 2015 | Y: P1.5-1,10-4: F4-1: ST-10294 (cc23) |  |  |
| 49783 | CM40873 | UK [England] | 2015 | Y: P1.5-2,10-2: F4-1: ST-183 (cc23) |  |  |
| 49797 | CM41001 | UK [England] | 2015 | Y: P1.5-1,2-2: F5-8: ST-23 (cc23) |  |  |
| 49817 | CM41330 | UK [England] | 2015 | Y: P1.5-2,10-2: F-ND: ST-183 (cc23) |  |  |
| 49855 | PL40423 | UK [England] | 2015 | Y: P1.5-1,10-1: F4-1: ST-1655 (cc23) |  |  |
| 49886 | PL40947 | UK [England] | 2015 | Y: P1.5-1,2-2: F5-8: ST-23 (cc23) |  |  |
| 49912 | PL41167 | UK [England] | 2015 | Y: P1.5-2,10-2: F5-8: ST-183 (cc23) |  |  |
| 49958 | PL41799 | UK [England] | 2015 | Y: P1.5-2,10-2: F4-1: ST-183 (cc23) |  |  |
| 49974 | PL42095 | UK [England] | 2015 | Y: P1.5-1,10-4: F4-1: ST-23 (cc23) |  |  |
| 50096 | ST41308 | UK [England] | 2015 | Y: P1.5-1,2-2: F5-8: ST-23 (cc23) |  |  |
| 50190 | WG41297 | UK [England] | 2015 | Y: P1.5-2,10-2: F1-7: ST-183 (cc23) |  |  |
| 50205 | WG41360 | UK [England] | 2015 | Y: P1.5-1,10-1: F4-1: ST-1655 (cc23) |  |  |
| 50301 | GL42561 | UK [Scotland] | 2015 | Y: P1.5-1,2-2: F5-8: ST-23 (cc23) |  |  |
| 50305 | GL42673 | UK [Scotland] | 2015 | Y: P1.5-1,2-2: F5-8: ST-23 (cc23) |  |  |
| 50306 | GL42674 | UK [Scotland] | 2015 | Y: P1.5-1,2-2: F5-8: ST-23 (cc23) |  |  |
| 50345 | ST41402 | UK [England] | 2015 | Y: P1.5-1,10-4: F4-1: ST-23 (cc23) |  |  |
| 50404 | ST42017 | UK [England] | 2015 | Y: P1.5-1,10-10: F4-1: ST-12353 (cc23) |  |  |
| 50437 | ST42326 | UK [England] | 2015 | Y: P1.5-1,10-4: F4-1: ST-23 (cc23) |  |  |
| 51229 | B119 | UK [England] | 2015 | Y: P1.5-2,10-29: F4-1: ST-23 (cc23) |  |  |
| 51247 | B189 | UK [England] | 2016 | Y: P1.5-2,10-2: F1-24: ST-23 (cc23) |  |  |
| 51255 | B208 | UK [England] | 2016 | Y: P1.5-1,10-1: F4-1: ST-12176 (cc23) |  |  |
| 51365 | R424 | UK [England] | 2015 | Y: P1.5-2,10-2: F4-1: ST-183 (cc23) |  |  |
| 51904 | 17-52 | Sweden | 2017 | Y: P1.5-1,2-2: F5-8: ST-23 (cc23) |  |  |
| 52561 | CA40083 | UK [Wales] | 2015 | Y: P1.5-2,10-2: F4-1: ST-183 (cc23) |  |  |
| 52571 | CA41303 | UK [Wales] | 2015 | Y: P1.7-2,4: F4-1: ST-2902 (cc23) |  |  |
| 52619 | GL41176 | UK [Scotland] | 2014 | Y: P1.5-2,10-2: F4-1: ST-183 (cc23) |  |  |
| 52641 | OX40264 | UK [England] | 2015 | Y: P1.5-1,10-4: F4-1: ST-23 (cc23) |  |  |
| 52853 | SMG_17_143 | UK [Scotland] | 2016 | Y: P1.5-1,2-2: F5-8: ST-6800 (cc23) |  |  |
| 53061 | M16 240718 | UK [England] | 2016 | Y: P1.5-1,10-4: F4-1: ST-23 (cc23) | >=65 | 2 |
| 53062 | M16 240719 | UK [England] | 2016 | Y: P1.5-1,10-1: F4-1: ST-1655 (cc23) | <65 | 1, 2 |
| 53066 | M16 240723 | UK [England] | 2016 | Y: P1.5-2,10-1: F5-8: ST-23 (cc23) | <65 | 1 |
| 53079 | M16 240742 | UK [England] | 2016 | Y: P1.5-1,10-1: F4-1: ST-1655 (cc23) | >=65 | 2, 3 |
| 53085 | M16 240749 | UK [England] | 2016 | Y: P1.5-1,10-1: F4-1: ST-13092 (cc23) | >=65 | 1, 2 |
| 53098 | M16 240767 | UK [England] | 2016 | Y: P1.5-1,10-1: F4-1: ST-1655 (cc23) | >=65 | 1, 2, 3 |
| 53103 | M16 240773 | UK [Wales] | 2016 | Y: P1.5-1,2-2: F5-8: ST-23 (cc23) | >=65 | 1, 2, 3 |
| 53106 | M16 240777 | UK [England] | 2016 | Y: P1.5-1,10-4: F4-1: ST-13093 (cc23) | >=65 | 1, 2, 3 |
| 53108 | M16 240779 | UK [England] | 2016 | Y: P1.5-1,10-4: F4-1: ST-1655 (cc23) | >=65 | 3 |
| 53112 | M16 240783 | UK [England] | 2016 | Y: P1.5-1,10-1: F4-1: ST-1655 (cc23) | <65 | 1 |
| 53122 | M16 240797 | UK [England] | 2016 | Y: P1.5-1,10-1: F4-1: ST-1655 (cc23) | >=65 | 2 |
| 53125 | M16 240803 | UK [England] | 2016 | Y: P1.5-1,10-1: F4-1: ST-1655 (cc23) | <65 | 3 |
| 53147 | M16 240840 | UK [England] | 2016 | Y: P1.5-1,10-1: F4-1: ST-1655 (cc23) | <65 | 2, 3 |
| 53155 | M16 240850 | UK [England] | 2016 | Y: P1.5-1,10-1: F4-1: ST-1655 (cc23) | <65 | 1, 2, 3 |
| 53158 | M16 240853 | UK [England] | 2016 | Y: P1.5-1,10-1: F4-1: ST-1655 (cc23) | <65 | 3 |
| 53162 | M16 240858 | UK [England] | 2016 | Y: P1.5-1,10-12: F4-1: ST-1655 (cc23) | <65 | 2, 3 |
| 53171 | M16 240870 | UK [England] | 2016 | Y: P1.5-1,10-1: F4-1: ST-1655 (cc23) | <65 | 1, 2, 3 |
| 53174 | M16 240875 | UK [England] | 2016 | Y: P1.5-1,10-1: F4-1: ST-1655 (cc23) | >=65 | 1, 3 |
| 53180 | M16 240882 | UK [England] | 2016 | Y: P1.5-1,10-1: F4-1: ST-1655 (cc23) | <65 | 2 |
| 53201 | M16 240909 | UK [England] | 2016 | Y: P1.5-2,10-1: F4-1: ST-23 (cc23) | >=65 | 1, 2 |
| 53202 | M16 240910 | UK [England] | 2016 | Y: P1.5-1,10-1: F4-1: ST-1655 (cc23) | >=65 | 1 |
| 53203 | M17 240001 | UK [England] | 2017 | Y: P1.5-1,10-1: F4-1: ST-1655 (cc23) | <65 | 2 |
| 53205 | M17 240003 | UK [England] | 2017 | Y: P1.7,10-1: F4-1: ST-6463 (cc23) | >=65 | 2, 3 |
| 53211 | M17 240009 | UK [England] | 2017 | Y: P1.5-2,10-1: F5-12: ST-23 (cc23) | >=65 | 1, 2 |
| 53218 | M17 240016 | UK [Wales] | 2017 | Y: P1.5-2,10-1: F4-1: ST-23 (cc23) | >=65 | 1, 3 |
| 53220 | M17 240018 | UK [England] | 2017 | Y: P1.5-1,10-105: F4-44: ST-23 (cc23) | <65 | 2 |
| 53227 | M17 240026 | UK [England] | 2017 | Y: P1.5-2,10-1: F5-12: ST-23 (cc23) | >=65 | 1, 2 |
| 53239 | M17 240038 | UK [England] | 2017 | Y: P1.5-1,10-1: F4-1: ST-1655 (cc23) | <65 | 1, 3 |
| 53245 | M17 240045 | UK [England] | 2017 | Y: P1.5-1,10-12: F4-1: ST-1655 (cc23) | >=65 | 2 |
| 53257 | M17 240059 | UK [England] | 2017 | Y: P1.5-1,10-1: F4-1: ST-1655 (cc23) | >=65 | 1, 2 |
| 53258 | M17 240062 | UK [England] | 2017 | Y: P1.5-1,10-1: F4-1: ST-1655 (cc23) | >=65 | 2, 3 |
| 53277 | M17 240088 | UK [England] | 2017 | Y: P1.5-1,10-1: F4-1: ST-1655 (cc23) | >=65 | 1, 2, 3 |
| 53283 | M17 240098 | UK [England] | 2017 | Y: P1.5-2,10-1: F5-12: ST-23 (cc23) | <65 | 1, 2, 3 |
| 53294 | M17 240125 | UK [England] | 2017 | Y: P1.5-1,10-1: F4-1: ST-1655 (cc23) | >=65 | 1, 3 |
| 53318 | M17 240154 | UK [England] | 2017 | Y: P1.5-1,10-1: F4-1: ST-12176 (cc23) | >=65 | 1, 2, 3 |
| 53324 | M17 240163 | UK [England] | 2017 | Y: P1.5-1,10-1: F4-1: ST-1655 (cc23) | <65 | 3 |
| 53330 | M17 240169 | UK [England] | 2017 | Y: P1.5-1,10-1: F4-1: ST-1655 (cc23) | >=65 | 1, 2, 3 |
| 53334 | M17 240174 | UK [England] | 2017 | Y: P1.5-1,10-1: F4-1: ST-1378 (cc23) | >=65 | 2 |
| 53353 | M17 240199 | UK [England] | 2017 | Y: P1.5-2,10-2: F2-13: ST-23 (cc23) | >=65 | 2 |
| 53720 | Nmiss2983 | Italy |  | Y: P1.5-2,10-2: F2-13: ST-13360 (cc23) |  |  |
| 53721 | NmissPE26 | Italy |  | Y: P1.5-2,ND: F2-13: ST-23 (cc23) |  |  |
| 53820 | LNP29202abd | France | 2017 | Y: P1.5-2,10-2: F4-1: ST-3587 (cc23) |  |  |
| 53931 | N222.3 | UK [England] | 2008 | Y: P1.5-1,10-1: F4-1: ST-1655 (cc23) |  |  |
| 53960 | N370.4 | UK [England] | 2009 | Y: P1.5-1,10-1: F4-1: ST-1655 (cc23) |  |  |
| 54483 | 17-292 | Sweden | 2017 | Y: P1.5-2,10-12: F5-9: ST-23 (cc23) |  |  |
| 54593 | LNP19808abd | France | 2002 | Y: P1.5-2,10-2: F4-1: ST-13355 (cc23) |  |  |
| 54607 | LNP21763abd | France | 2004 | Y: P1.5-2,10-2: F2-13: ST-23 (cc23) |  |  |
| 56082 | NM03010 | USA | 2006 | Y: P1.5-2,10-1: F4-1: ST-23 (cc23) |  |  |
| 56111 | NM03040 | USA | 2006 | Y: P1.5-2,10-1: F4-1: ST-23 (cc23) |  |  |
| 56112 | NM03041 | USA | 2007 | Y: P1.5-1,2-2: F5-8: ST-23 (cc23) |  |  |
| 56117 | NM03047 | USA | 2007 | Y: P1.5-2,10-1: F4-1: ST-23 (cc23) |  |  |
| 56123 | NM03053 | USA | 2007 | Y: P1.5-2,10-1: F4-1: ST-13353 (cc23) |  |  |
| 56419 | Men20F1 | UK | 2017 | Y: P1.5-2,10-2: F2-13: ST-23 (cc23) |  |  |
| 58163 | 17-495 | Sweden | 2017 | Y: P1.5-2,10-1: F4-1: ST-4183 (cc23) |  |  |
| 59102 | NM01909 | USA | 2006 | Y: P1.5-2,10-2: F4-1: ST-183 (cc23) |  |  |
| 59106 | NM01916 | USA | 2006 | Y: P1.5-2,10-1: F4-1: ST-23 (cc23) |  |  |
| 59112 | NM01926 | USA | 2006 | Y: P1.5-2,10-2: F4-1: ST-183 (cc23) |  |  |
| 59113 | NM01928 | USA | 2006 | Y: P1.5-2,10-2: F4-1: ST-183 (cc23) |  |  |
| 59117 | NM01935 | USA | 2006 | Y: P1.5-2,10-1: F4-1: ST-23 (cc23) |  |  |
| 59119 | NM01937 | USA | 2006 | Y: P1.5-2,10-2: F4-1: ST-183 (cc23) |  |  |
| 59120 | NM01939 | USA | 2006 | Y: P1.5-2,10-2: F4-1: ST-183 (cc23) |  |  |
| 59153 | NM02496 | USA | 2006 | Y: P1.5-2,10-1: F4-1: ST-23 (cc23) |  |  |
| 59154 | NM02497 | USA | 2006 | Y: P1.5-2,10-1: F4-1: ST-23 (cc23) |  |  |
| 59165 | NM02517 | USA | 2006 | Y: P1.5-2,10-2: F4-1: ST-183 (cc23) |  |  |
| 59170 | NM02527 | USA | 2006 | Y: P1.5-2,10-2: F4-1: ST-183 (cc23) |  |  |
| 59171 | NM02529 | USA | 2006 | Y: P1.5-2,10-2: F4-1: ST-183 (cc23) |  |  |
| 59176 | NM02539 | USA | 2006 | Y: P1.5-2,10-2: F4-1: ST-183 (cc23) |  |  |
| 59180 | NM02547 | USA | 2006 | Y: P1.5-2,10-2: F4-1: ST-183 (cc23) |  |  |
| 59190 | NM02565 | USA | 2006 | Y: P1.5-2,10-1: F4-1: ST-23 (cc23) |  |  |
| 59211 | NM02608 | USA | 2007 | Y: P1.5-2,10-1: F4-1: ST-23 (cc23) |  |  |
| 59213 | NM02612 | USA | 2007 | Y: P1.5-2,10-1: F4-1: ST-23 (cc23) |  |  |
| 59214 | NM02615 | USA | 2007 | Y: P1.5-2,10-1: F4-1: ST-23 (cc23) |  |  |
| 59217 | NM02619 | USA | 2007 | Y: P1.5-2,10-1: F4-1: ST-23 (cc23) |  |  |
| 59222 | NM02628 | USA | 2007 | Y: P1.5-2,10-1: F4-1: ST-23 (cc23) |  |  |
| 59225 | NM02634 | USA | 2007 | Y: P1.5-2,10-2: F4-1: ST-183 (cc23) |  |  |
| 59236 | NM02659 | USA | 2007 | Y: P1.5-2,10-2: F4-1: ST-183 (cc23) |  |  |
| 59237 | NM02661 | USA | 2007 | Y: P1.5-2,10-2: F4-1: ST-183 (cc23) |  |  |
| 59241 | NM02669 | USA | 2007 | Y: P1.5-2,10-2: F4-1: ST-183 (cc23) |  |  |
| 59252 | NM02693 | USA | 2007 | Y: P1.5-2,10-1: F4-1: ST-23 (cc23) |  |  |
| 59261 | NM02714 | USA | 2007 | Y: P1.5-2,10-1: F4-1: ST-4245 (cc23) |  |  |
| 59270 | NM02733 | USA | 2007 | Y: P1.5-1,2-2: F5-8: ST-23 (cc23) |  |  |
| 59292 | NM02767 | USA | 2007 | Y: P1.5-1,2-2: F5-8: ST-6799 (cc23) |  |  |
| 59295 | NM02770 | USA | 2007 | Y: P1.5-1,2-2: F5-8: ST-23 (cc23) |  |  |
| 59297 | NM02772 | USA | 2007 | Y: P1.5-1,2-2: F5-8: ST-23 (cc23) |  |  |
| 59305 | NM02977 | USA | 2007 | Y: P1.5-2,10-1: F4-1: ST-23 (cc23) |  |  |
| 59403 | NM03080 | USA | 2007 | Y: P1.5-1,2-2: F5-8: ST-23 (cc23) |  |  |
| 59421 | NM03098 | USA | 2007 | Y: P1.5-2,10-1: F4-1: ST-23 (cc23) |  |  |
| 59422 | NM03099 | USA | 2007 | Y: P1.5-2,10-1: F4-1: ST-23 (cc23) |  |  |
| 59441 | NM03119 | USA | 2007 | Y: P1.5-1,2-2: F5-8: ST-23 (cc23) |  |  |
| 59452 | NM03131 | USA | 2008 | Y: P1.5-1,2-2: F5-8: ST-893 (cc23) |  |  |
| 59696 | 48550 | South Africa | 2016 | Y: P1.5-2,10-1: F4-1: ST-13846 (cc23) |  |  |
| 59699 | 48758 | South Africa | 2016 | Y: P1.5-1,2-2: F5-8: ST-6800 (cc23) |  |  |
| 59704 | 49119 | South Africa | 2016 | Y: P1.5-2,10-1: F4-1: ST-4245 (cc23) |  |  |
| 59721 | 51460 | South Africa | 2017 | Y: P1.5-2,10-1: F4-1: ST-4245 (cc23) |  |  |
| 59829 | 52763 | South Africa | 2017 | Y: P1.5-2,10-1: F4-1: ST-4245 (cc23) |  |  |
| 59888 | C10686 | South Africa | 2017 | Y: P1.5-1,2-2: F5-8: ST-6800 (cc23) |  |  |
| 59892 | W10882 | South Africa | 2017 | Y: P1.5-2,10-1: F4-1: ST-4245 (cc23) |  |  |
| 59925 | C20354 | South Africa | 2017 | Y: P1.5-1,10-1: F4-1: ST-1655 (cc23) |  |  |
| 59926 | C20367 | South Africa | 2017 | Y: P1.5-1,2-2: F5-8: ST-6800 (cc23) |  |  |
| 59931 | C20648 | South Africa | 2017 | Y: P1.5-1,10-1: F4-1: ST-1655 (cc23) |  |  |
| 59945 | C20749 | South Africa | 2017 | Y: P1.5-1,10-1: F4-1: ST-1655 (cc23) |  |  |
| 60848 | BRI018 | UK | 2017 | Y: P1.5-1,2-2: F5-8: ST-23 (cc23) |  |  |
| 61190 | 52105 | South Africa | 2017 | Y: P1.5-2,10-1: F4-1: ST-4245 (cc23) |  |  |
| 61193 | C20259 | South Africa | 2017 | Y: P1.5-1,10-1: F4-1: ST-1655 (cc23) |  |  |
| 61295 | 18-161 | Sweden | 2018 | Y: P1.5-1,2-2: F5-8: ST-23 (cc23) |  |  |
| 61303 | 18-215 | Sweden | 2018 | Y: P1.5-2,10-1: F4-1: ST-23 (cc23) |  |  |
| 61308 | 18-296 | Sweden | 2018 | Y: P1.5-1,10-4: F4-1: ST-1655 (cc23) |  |  |
| 61958 | Bris010 | UK | 2014 | Y: P1.5-1,2-2: F5-8: ST-23 (cc23) |  |  |
| 62995 | SMG-18-1776 | UK [Scotland] | 2018 | Y: P1.5-1,10-1: F4-1: ST-1655 (cc23) |  |  |
| 83690 | C1 0567 | South Africa | 2017 | ND: P1.5-1,10-1: F4-1: ST-1655 (cc23) |  |  |
| 83691 | W10673 | South Africa | 2017 | Y: P1.5-1,10-1: F4-1: ST-1655 (cc23) |  |  |
| 84111 | 18-375 | Sweden | 2018 | Y: P1.5-2,10-1: F4-1: ST-23 (cc23) |  |  |
| 84117 | 18-411 | Sweden | 2018 | Y: P1.5-2,10-1: F4-1: ST-23 (cc23) |  |  |
| 84125 | 18-607 | Sweden | 2018 | Y: P1.5-1,2-2: F5-8: ST-23 (cc23) |  |  |
| 84898 | DE13997 | Germany | 2017 | Y: P1.5-1,10-4: F4-1: ST-23 (cc23) |  |  |

**Supplementary Table 3** Highest scoring genes in Scoary analysis of 100 isolates aged under 65 and 100 isolates aged over 65.

**Supplementary table 4** Loci within serogroup Y cc23 strains reaching significance (p<0.0001) in three TreeWas analyses, including number of non-synonymous single nucleotide polymorphisms identified in each analysis and the lowest p value at each locus (*tbpB* identified in each analysis – highlighted)

| **Analysis 1** | | | | **Analysis 2** | | | | **Analysis 3** | | | |
| --- | --- | --- | --- | --- | --- | --- | --- | --- | --- | --- | --- |
| **Loci** | **Total number SNPs identified** | **Number non-synonymous SNPs** | **p-value** | **Loci** | **Total number SNPs identified** | **Number non-synonymous SNPs** | **p-value** | **Loci** | **Total number SNPs identified** | **Number non-synonymous SNPs** | **p-value** |
| NEIS0361 – hypothetical protein | 1 | 0 | 6.05x10^-5^ | NEIS0221 – MafI immunity protein (*mafI_o1MGI-1_)* | 17 | 7 | 2.64x10^-5^ | NEIS0033 – type IV pilus associated protein, *(pilC2)* | 1 | 1 | 5.17x10^-5^ |
| NEIS1690 – transferrin binding protein A (*tbpA)* | 1 | 0 | 6.05x10^-5^ | **NEIS1691 – transferrin binding protein B, (*tbpB)*** | **10** | **6** | **3.30x10^-5^** | NEIS0597 – MafB1/MafB2 toxin protein *(mafB_MGI-1_ or mafB2_MGI-2_)* | 1 | 0 | 8.47x10^-5^ |
| **NEIS1691 – transferrin binding protein B, (*tbpB)*** | **4** | **2** | **5.2x10^e-5^** |  |  |  |  | NEIS1690 – transferrin binding protein A *(tbpA)* | 1 | 0 | 7.26x10^-5^ |
| NEIS1440 – ribose-5-phosphate isomerase A, (*rpiA*) | 5 | 1 | 5.82x10^-5^ |  |  |  |  | **NEIS1691 – transferrin binding protein B *(tbpB)*** | **20** | **16** | **5.50x10^-6^** |
| NEIS1441 – part of methylerythritol 4-phosphate (MEP) pathway | 1 | 1 | 5.25x10^-5^ |  |  |  |  |  |  |  |  |
| NEIS1442 – part of methylerythritol 4-phosphate (MEP) part of MEP pathway | 3 | 0 | 5.82x10^-5^ |  |  |  |  |  |  |  |  |
| NEIS1727 – acetate kinase (*ackA2*) | 13 | 1 | 6.74x10^-5^ |  |  |  |  |  |  |  |  |
